# Macrophage-associated kinase signaling in atherosclerosis – a systematic review

**DOI:** 10.1186/s12964-026-02829-9

**Published:** 2026-03-27

**Authors:** Jana S. Müller, Emiel P. C. van der Vorst

**Affiliations:** 1 Department of Internal Medicine I, Aachen-Maastricht Institute for Cardio-Renal Disease (AMICARE), Institute for Molecular Cardiovascular Research (IMCAR), University Hospital Aachen, RWTH Aachen University, Aachen, Germany; 2 Institute for Cardiovascular Prevention (IPEK), Ludwig-Maximilians-Universität, Munich, Germany

**Keywords:** Atherosclerosis, Macrophages, Kinases, Inflammation, Therapy

## Abstract

Atherosclerosis represents a chronic inflammatory disease of the arterial wall and remains a principal cause of cardiovascular morbidity and mortality. Macrophages critically govern lesion initiation, progression, and destabilization, and accumulating evidence indicates that protein kinases are key regulators of their phenotype and function.

This systematic review synthesizes data from 162 publications encompassing 76 kinases to delineate the contribution of macrophage-associated kinase signaling to atherogenesis. The identified kinases span major families, including AGC, CaMK, CMGC, Ste20, and tyrosine kinases, each exerting distinct regulatory effects on macrophage survival, polarization, lipid handling, efferocytosis, and inflammatory activation. Several kinases, such as CaMK2γ, CaMK4, DCLK1, Trib1, and STK25, exhibit pro-atherogenic activity by promoting foam cell formation, expanding the necrotic core, and propagating inflammatory pathways. Conversely, kinases, including STK11 and the context-dependent mediator Akt1, exhibit protective or dual functions that contribute to metabolic homeostasis and reparative macrophage states. Despite substantial mechanistic insights and the established therapeutic utility of kinase inhibitors in oncology, clinical translation in the context of atherosclerosis remains limited.

This review consolidates current knowledge, identifies critical gaps, and outlines prospective avenues to target macrophage-specific kinase pathways as novel therapeutic strategies for atherosclerosis.

## Background

Atherosclerosis is a common cause of cardiovascular disease (CVD), such as coronary heart disease or stroke. According to the World Health Organization (WHO), around 32% of all global deaths were related to CVD in 2022, and these numbers are still rising. The high mortality rate is accompanied by an enormous disease burden that requires medical treatment. That is why CVD, and in particular atherosclerosis, have become increasingly important in medical research [1].

**Fig. 1:**
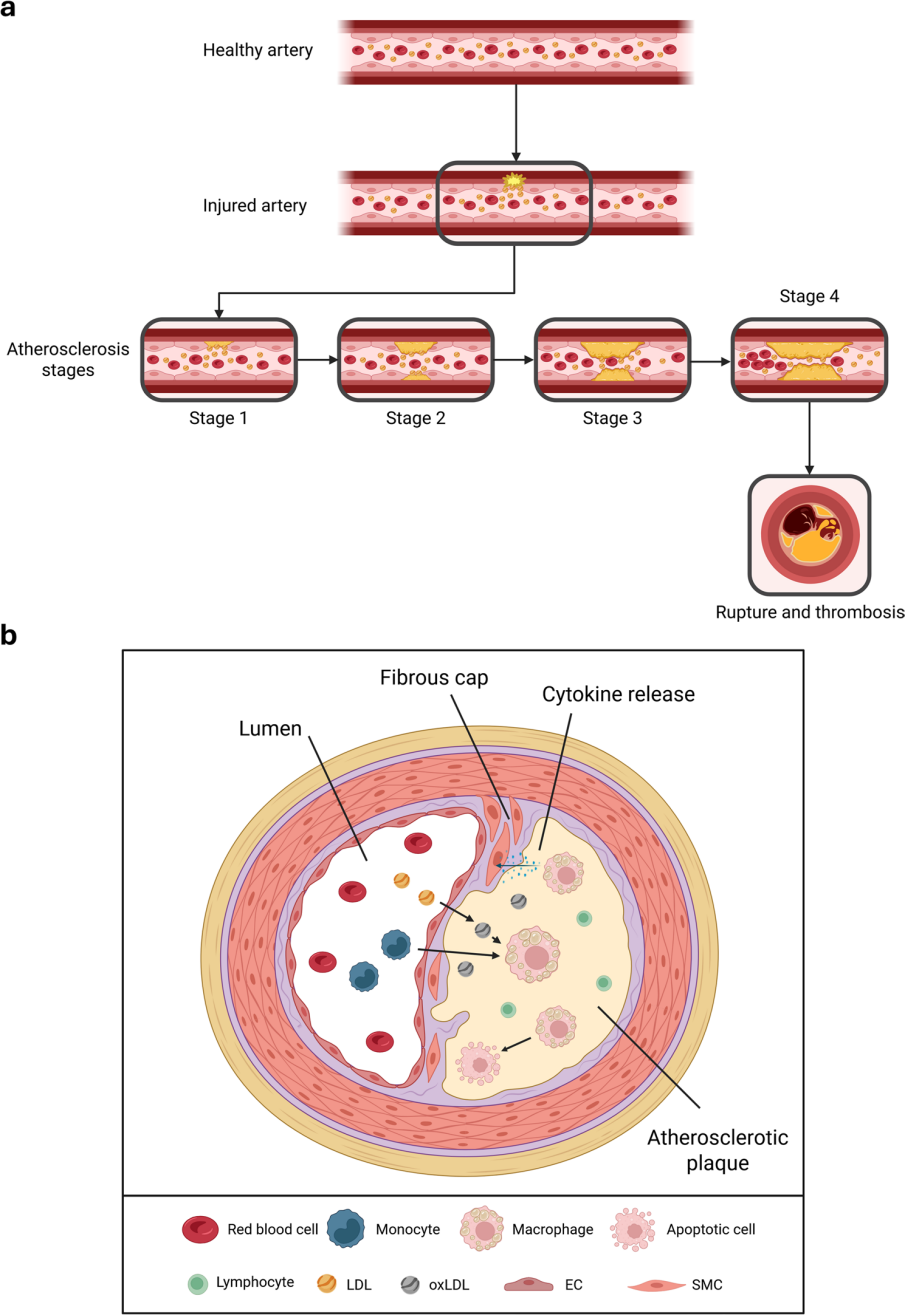
Schematic overview of the development and cellular composition of an atherosclerotic plaque. **a** Illustration of the stepwise progression from a healthy artery to early endothelial dysfunction, lipid accumulation, and formation of fatty streaks, followed by plaque maturation. Continued plaque growth leads to the formation of a necrotic core and the development of advanced lesions. **b **Depiction of a cross-section of an established atherosclerotic plaque, highlighting key structural features (e.g., fibrous cap, lipid-rich necrotic core, and vascular smooth muscle cell layer), as well as the major involved cell types. EC: endothelial cell; LDL: low-density lipoprotein; oxLDL: oxidized LDL; SMC: smooth muscle cell

High blood pressure, diabetes, smoking, and high levels of low-density lipoprotein (LDL) cholesterol are general risk factors for developing atherosclerotic plaques and thereby CVD [2]. The endothelial layer of large arteries becomes damaged or inflamed, resulting in the accumulation of LDL in the intima, where it is oxidized (oxLDL) and triggers further inflammatory processes [2, 3]. Increased expression of adhesion molecules, such as vascular cell adhesion molecule-1 (VCAM-1) and intercellular adhesion molecule-1 (ICAM-1), facilitates leukocyte migration, particularly monocytes, into the intima [4, 5]. Monocytes differentiate into macrophages, which then take up the present oxLDL via scavenger receptors (SR), such as SR-A or cluster of differentiation 36 (CD36). The absorbed oxLDL converts macrophages into foam cells, which continue to accumulate lipids and eventually die by apoptosis or secondary necrosis. Dead macrophages form the lipid-rich, soft core of the plaques [2, 6, 7], which is further stabilized through migrated smooth muscle cells (SMCs) into the intima [8]. There, the SMCs proliferate and form collagen-rich structures known as the fibrous cap [9]. Over time, a chronic lipid load leads to inflammatory destruction, resulting in thin, destabilized fibrous caps that eventually rupture or erode, releasing the thrombogenic core [10]. This can cause an acute vascular occlusion, which can, depending on the location, result in a heart attack or stroke (Fig. 1) [11]. Besides plaque rupture, recent studies have identified another process that leads to acute coronary syndromes predominantly affecting women, smokers, and younger patients, accounting for up to 40% of cases: plaque erosion, which occurs in the presence of an intact fibrous cap. These plaques are characterized by a proteoglycan- and vascular SMC-rich composition, with low lipid content and minimal macrophage infiltration. Plaque erosion is initiated by endothelial dysfunction or endothelial loss induced by disturbed shear stress, leading to innate immune activation, neutrophil recruitment, and the formation of platelet-rich thrombi, often in a low-grade inflammatory environment [12].

One of the crucial regulators of macrophage function and, thereby, atherosclerosis development is the kinase family. Kinases phosphorylate proteins using adenosine triphosphate (ATP) and are essential in cell-cell communication as well as in cell division, cell growth, signal transduction, protein synthesis, apoptosis, and are also responsible for the M1-M2-Macrophage balance [13]. While M1-macrophages promote pro-inflammatory processes and lead to plaque instability in atherosclerosis, M2-macrophages reduce inflammation, induce tissue repair and wound healing, and stabilize atherosclerotic plaques [14, 15]. Typical kinase signaling pathways in macrophages are the JAK/STAT, NF-κB, and the PI3K/Akt/mTOR pathway. While the JAK/STAT pathway is the central axis for macrophage polarization, NF-κB is a key hub of innate immune activation, and PI3K/Akt/mTOR is a major regulator of macrophage survival, metabolism, migration, and metabolic reprogramming [16]. Although the roles of many kinases in atherosclerosis have been studied, no therapeutic options for this condition are currently in clinical use. In contrast, in oncology, specific kinase inhibitors such as imatinib, sunitinib, and erlotinib have already been developed for the targeted treatment of malignant tumors and are in clinical use [17, 18]. This highlights the clinical potential of kinase targeting, opening new avenues for kinase-related therapeutic approaches to combat atherosclerosis and CVD.

Therefore, we conducted a systematic review of the current literature on the role of macrophage-related kinases in atherosclerosis, providing broad insight into the complex signaling network and potential therapeutic targets, and highlighting current limitations and knowledge gaps.

## Methods

### Paper selection

This systematic review is based on a selection of articles in PubMed (July 23, 2025) regarding atherosclerosis and macrophages in relation to humans, as well as the official kinase list of the ProteinAtlas website [19] and the classification of kinases into their respective kinase families using the KinBase.com website [20].

The literature was selected on PubMed in three runs according to specific search terms beginning with “atherosclerosis AND macrophage AND Human”, further extended to “atherosclerosis AND macrophage AND Human AND (*kinase name*)”, and finally specialized to “atherosclerosis AND macrophage AND Human AND (clinical OR therapeutic OR therapeutical) AND (kinase name)”. The used kinase names were selected from the Protein Atlas [19] using the search term “protein_class: kinases” and were added one after the other based on the corresponding terms.

**Fig. 2:**
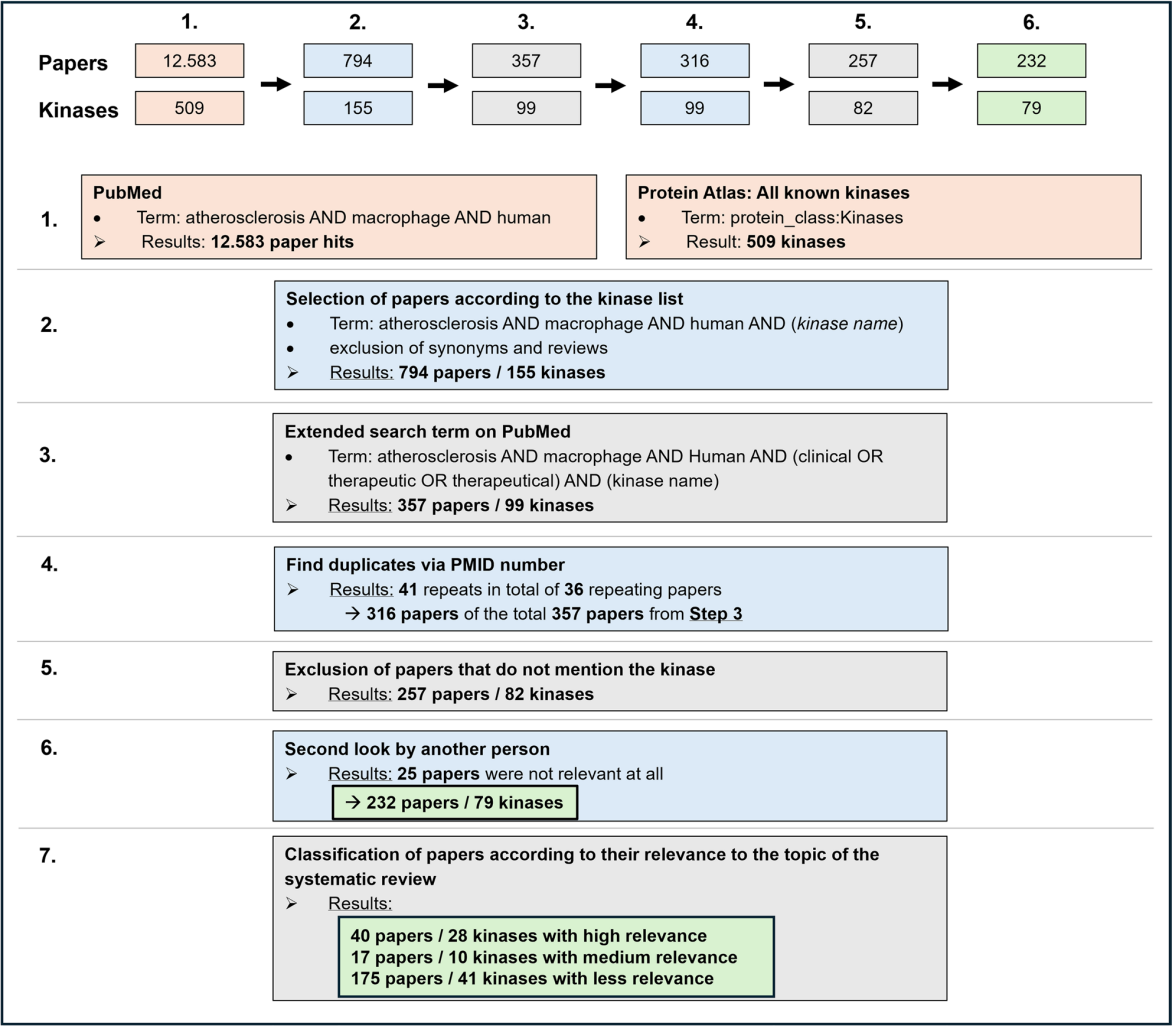
Workflow for the systematic identification and selection of kinase-related literature in human atherosclerosis. Beginning with an initial PubMed search for studies on atherosclerosis, macrophages, and humans (12.583 hits), and a complementary kinase gene list obtained from the Protein Atlas (509 kinases), a stepwise filtering strategy was applied. Papers were screened using kinase-specific search terms, extended clinical/therapeutic filters, and duplicate removal by PMID. Subsequent exclusion of studies that did not mention the kinase, along with secondary manual validation, yielded 232 papers covering 79 kinases. Finally, the included studies were categorized as high, medium, or low in relevance to the systematic review

The first run yielded 12.583 papers on PubMed and identified 509 existing kinases listed on ProteinAtlas [19] (Fig. 2). The second narrowed the scope of the literature to 794 papers across 155 kinases, and the third narrowed it to 357 papers and 99 kinases. In a fourth step, duplicates were identified and removed from the dataset, yielding 316 papers for 99 kinases. In the fifth step, all papers that did not mention the kinase at all, for example, due to ambiguous kinase names, such as KIT or MET, were excluded, narrowing the scope to 257 papers and 82 genes. The following step, conducted by a second independent person, excluded 25 additional papers: those published before 2000, those available only in a foreign language, or those with no access. The remaining literature collection, comprising 175 papers for 79 kinases, was further divided into three groups according to their relevance to the topic of this systematic review: 40 papers were deemed highly relevant for 28 kinases (primarily addressing a kinase in the context of atherosclerosis), 17 were considered medium relevant for 10 kinases (secondary focus on a kinase in the context of atherosclerosis), and 175 were considered less relevant for 41 kinases (kinase merely mentioned as part of a signaling cascade or in a related context) (Fig. 2).

### Kinase categorization

The kinase family comprises 509 individual members [19]. These kinases can be functionally distinguished by the proteins they phosphorylate. That is why they are often described as serine/threonine and tyrosine kinases. According to the standard kinase classification system established by Manning et al., however, the categorization of individual kinases is primarily based on the characteristics of their catalytic eukaryotic protein kinase (ePK) domain, their phylogenetic relationships, and structural features outside the catalytic core, such as additional regulatory or binding domains. In addition to structural criteria, this classification also considers biological functions, including signaling pathways, substrate specificities, and regulatory mechanisms. Comparative analyses with model organisms, such as yeast, worms, or flies, are further used to identify conserved kinase groups [21, 22].

**Fig. 3:**
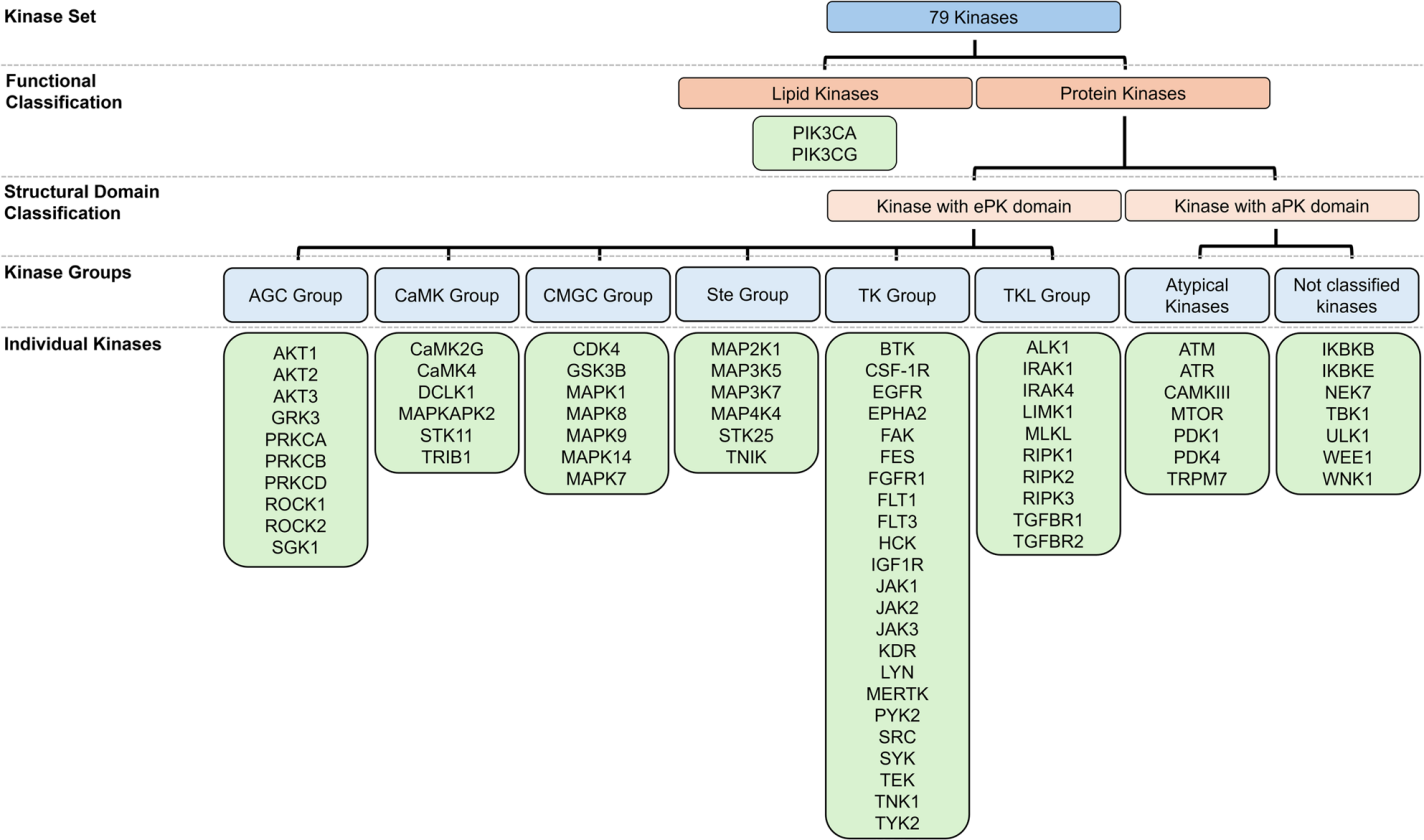
Classification of the 79 kinases identified in the systematic review. Kinases implicated in human atherosclerosis and macrophage biology are grouped by major structural families, including lipid and protein kinases, and further subdivided into AGC, CaMK, CMGC, Ste, TK, TKL, atypical, and unclassified families. Individual kinase names listed beneath each subgroup represent those for which relevant literature was identified. ePK: eukaryotic protein kinase; aPK: atypical protein kinase

All the kinases identified as associated with atherosclerosis and macrophages, as determined by the selected search criteria, are listed in Table 1, along with the number of paper hits (not excluding double hits) and their subsequent classification according to the paper’s relevance to this systematic review [20]. Furthermore, the listed kinases can be categorized after Manning et al. [22], as shown in Fig. 3.

**Table 1:** Overview of kinase groups, individual kinases, and the number of publications identified, categorized by relevance to the systematic review

Group	Kinase	Paper hits			
		Total	highly relevant	relevant	less relevant
AGC Group	AKT1	6	2		4
	AKT2	1			1
	AKT3	1			1
	GRK3	1			1
	PRKCA	2			2
	PRKCB	1			1
	PRKCD	1	1		
	ROCK1	1			1
	ROCK2	2			2
	SGK1	1			1
CaMK Group	CaMK2G	1		1	
	CaMK4	1	1		
	DCLK1	1	1		
	MAPKAPK2	1			1
	STK11	2		1	1
	TRIB1	1	1		3
CMGC Group	CDK4	1			1
	GSK3B	1			1
	MAPK1	1	1		
	MAPK8 (JNK1)	4			5
	MAPK9 (JNK2)	2			55
	MAPK14	1		1	
	MAPK7	1			1
STE Group	MAP2K1	1			1
	MAP3K5	1			1
	MAP3K7	1			1
	MAP4K4	1	1		
	STK25	1	1		
	TNIK	1		1	
TK Group	BTK	4	2		2
	CSF-1R	7		1	6
	EGFR	4	1	2	1
	EPHA2	2	2		
	FAK	1		1	
	FES	1	1		
	FGFR1	1	1		
	FLT1	2			2
	FLT3	1			1
	HCK	1			1
	IGF1R	1			1
	JAK1	5			5
	JAK2	13	2	1	10
	JAK3	1			1
	KDR	1			1
	LYN	2		1	2
	MerTK	13	1		12
	PYK2	1			1
	SRC	9			9
	SYK	7	2		5
	TEK	1			1
	TNK1	1		1	
	TYK2	4			4
TKL Group	ALK1	1	1		
	IRAK1	3		1	2
	IRAK4	2		2	
	LIMK1	5		0	5
	MLKL	2			2
	RIPK1	3	1		2
	RIPK2	1	1		
	RIPK3	6		1	5
	TGFBR1	2			2
	TGFBR2	1			1
Atypical Kinases	ATM	1			1
	ATR	1			1
	CaMKIII	1	1		
	MTOR	28	3	2	23
	PDK1	2	1		1
	PDK4	2			2
	TRPM7	1			1
Not Classified	IKBKB	5	2	1	2
	IKBKE	1	1		
	NEK7	1			1
	TBK1	2			2
	ULK1	2			2
	WEE1	1	1		
	WNK1	1			1
Lipid Kinases	PIK3CA	2			2
	PIK3CG	1			1

## Results

To structure the information on the role of macrophage-related kinases in atherosclerosis, the results are divided into kinase groups.

### AGC group

The protein kinase A/G/C related group (AGC) is named after its three primary family members: PKA (cAMP-dependent), PKG (cGMP-dependent), and PKC (calcium- and diacylglycerol-dependent). This group comprises more than 60 kinases and is characterized by a conserved catalytic domain that creates the ATP-binding site. AGC kinases are mainly involved in cell growth, survival, and differentiation, as well as in the cell cycle, proliferation, cell migration, cytoskeletal organization, metabolism, and signal transduction. Consequently, this group is associated with various pathological processes, including cancer, diabetes, inflammation, neurological disorders, and CVD [23].

**Fig. 4:**
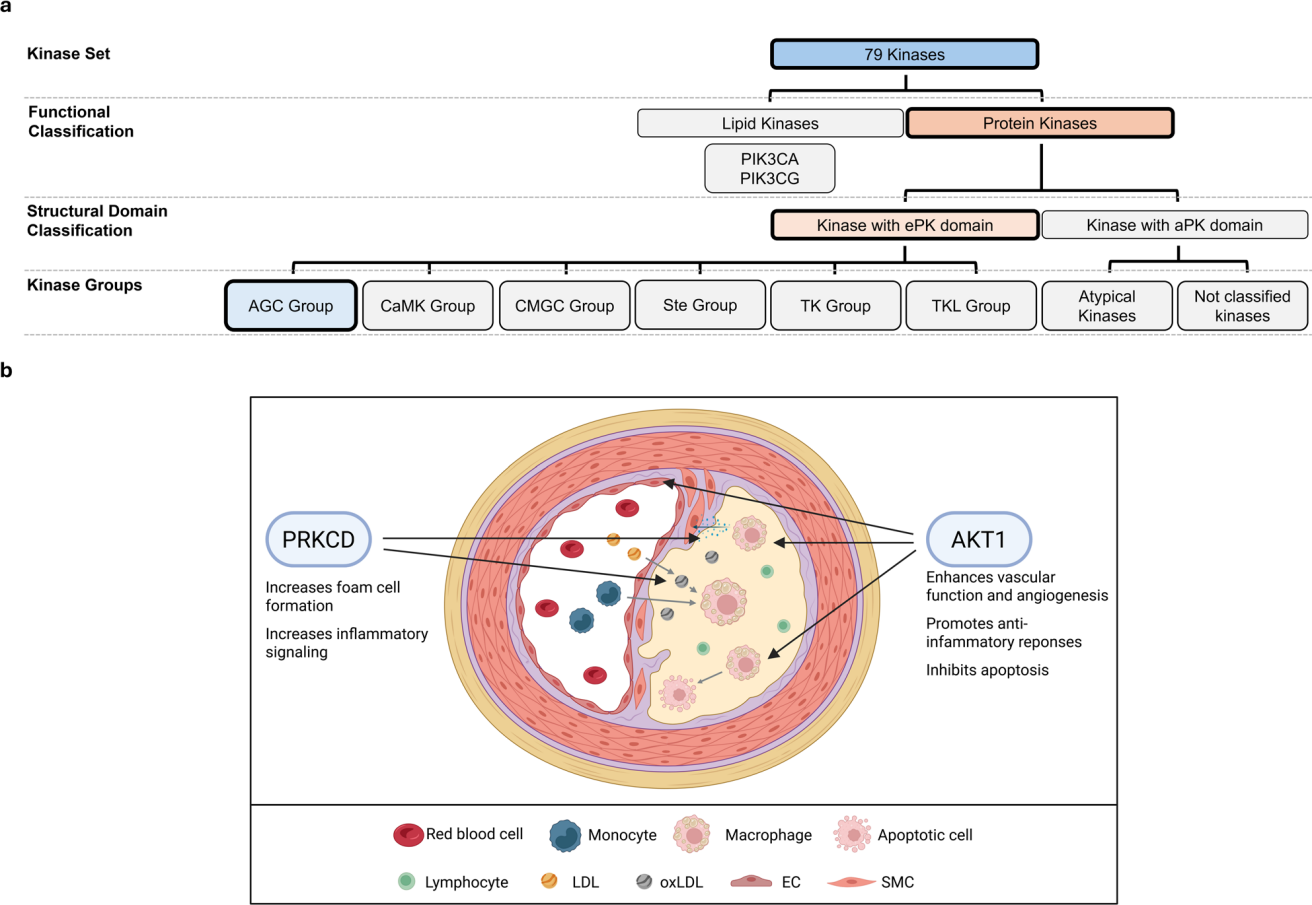
Overview of the role of the AGC Group of kinases in atherosclerosis. **a** Classification of the 76 kinases into major structural and functional groups, highlighting the AGC Group. **b** Illustration of the cellular microenvironment of an atherosclerotic lesion and visualizing the involvement of AGC Group kinases. EC: endothelial cell; LDL: low-density lipoprotein; oxLDL: oxidized LDL; SMC: smooth muscle cell

The AKT1 and PRKCD kinases have already been shown to be directly involved in atherosclerosis [24–28] and are discussed in detail below. In contrast, the kinases AKT2, AKT3, GRK3, PRKCA/PRKCB, ROCK1/ROCK2, and SGK1 have so far only been implicated in the disease, primarily through their regulatory roles in macrophage activation, inflammation, and migration [28–33] (Fig. 4).

#### AKT1

The AKT serine/threonine protein kinases comprise three different isoforms: AKT1, AKT2, and AKT3. They regulate a wide range of cellular processes in response to phosphoinositide 3-kinase (PI3K), including cell metabolism, survival, migration, and gene expression. Furthermore, these kinases have an essential role within the vascular wall [34, 35], by regulating the proliferation and migration of endothelial cells (ECs), controlling vascular permeability, and influencing angiogenesis [36–38]. Among the three isoforms, AKT1 is the primary isoform in macrophages and dendritic cells [39], where it promotes cell survival, growth, and differentiation. Moreover, it is found in SMCs, monocytes [25], platelets [37], and cardiac tissue, thereby contributing to the regulation of the cardiovascular system through multiple signaling pathways [25, 26, 40–42].

Moreover, the macrophage 1/macrophage 2 (M1/M2) polarization depends on AKT1. While the deficiency of AKT1 is associated with an enhanced pro-inflammatory response characterized by increased M1 polarization, the presence of this kinase promotes anti-inflammatory signaling through the induction of M2 polarization [25, 26, 43, 44]. Nevertheless, the kinase AKT1 displays a Janus-faced role in CVD, dependent on its surrounding conditions [27, 45–47]. For example, it has been shown that loss of *Akt1* in high-cholesterol diet (HCD)-apolipoprotein E (*Apoe)*^*−/−*^ mice led to an increased atherosclerosis development [26]. In *Apoe*^*−/−*^*Akt1*^*−/−*^ mice, vascular inflammation was also markedly increased, reflected by elevated expression of pro-inflammatory mediators such as interleukin-6 (IL-6), tumor necrosis factor (TNF), and VCAM-1, along with reduced levels of the antiangiogenic factor thrombospondin-1 (THBS1). This inflammatory state was further characterized by increased macrophage infiltration and reduced endothelial nitric oxide synthase (eNOS) phosphorylation, indicating pronounced endothelial dysfunction [26].

While *Akt1* deficiency markedly affected EC behavior in vivo and in vitro, bone marrow-derived macrophages (BMDMs) exhibited no significant functional alterations, maintaining normal adhesion, spreading, and chemokine-driven migration, as well as unaltered uptake of modified lipoproteins and foam cell formation upon exposure to oxidized low-density lipoprotein (oxLDL) [26]. In contrast, peritoneal macrophages from *Apoe*^*−/−*^*Akt1*^*−/−*^ mice were more susceptible to apoptosis induced by oxLDL or free cholesterol loading than those from control *Apoe*^*−/−*^*-*mice [26]. However, *Akt1*^*−/−*^ macrophages are not responsible for the enhanced atherosclerosis, as lethally irradiated *Apoe*^*−/−*^ recipient mice transplanted with bone marrow from either genotype showed no differences in lesion development. These findings indicate that vascular dysfunction of the host vessel wall, rather than defects in myeloid cells such as macrophages, is likely responsible for the aggravated atherosclerotic phenotype [26].

While these results confirm the more protective effects of AKT1 in atherosclerosis, another study [25] highlights the detrimental effects of sustained AKT1 activation under dyslipidemic stress conditions. Therefore, double knockout (dKO) Scavenger-receptor class B type I *(Srb1)*^−/−^*Apoe*^*−/−*^ mice and triple knockout (tKO) *Apoe*^*−/−*^*Srb1*^*−/−*^*Akt1*^*−/−*^ mouse models were compared [25]. *Srb1*^−/−^*Apoe*^*−/−*^ mice, exhibiting severe dyslipidemia and following myocardial infarction (MI), there was a correlation between activated AKT1 and cardiac dysfunction, hypertrophy, and fibrosis, as demonstrated by the finding that *Akt1* deletion improves lifespan and fitness under dyslipidemia and reduces infarct damage. Furthermore, *Akt1* deficiency reduced apoptosis and cardiac damage by decreasing fibrosis and necrosis and significantly reduced atherosclerotic lesion area and coronary plaque burden. Consistently, chronic Akt1 activation was shown to enhance oxidative stress-driven lipid oxidation and to increase CD36 expression on macrophages, thereby promoting CD36-dependent oxLDL uptake and foam cell formation, effects that were largely reversed by Akt1 deletion. Nevertheless, cholesterol levels remain unchanged between the dKO and tKO, *Apoe*^*−/−*^, and *Apoe*^*−/−*^*Akt1*^*−/−*^ mouse models, indicating that the anti-atherosclerotic effect is not a metabolic effect but a cellular one [25]. Mechanistically, these changes are embedded in a PI3K-Akt1-GSK3 axis, with elevated phosphorylation of AKT1 and GSK3 in dKO hearts and aortas, which is markedly reduced in tKO animals. In addition, in an independent coronary ligation MI model, global *Akt1* deletion reduced infarct size several weeks after ligation, supporting a broader benefit of limiting sustained AKT1 signaling under ischemic stress.

Furthermore, in dKO-hearts, p-IKKα/β levels were 8-fold higher than in WT mice, indicating that the nuclear factor kappa-light-chain-enhancer of activated B cells (NF-κB) signaling pathway was strongly activated. In comparison, the p-IKKα/β-levels were 1.9-fold lower in tKO mice. Furthermore, tKO mice exhibited reduced oxidative stress and lipid peroxide levels. The reduced amount of reactive oxygen species (ROS) upon *Akt* deficiency was confirmed in isolated ECs and macrophages from WT, dKO, and tKO. Simultaneously, oxLDL treatment of these cell types resulted in reduced oxLDL-induced apoptosis in the absence of *Akt1* [25]. A key concept is an AKT1-ROS-CD36 feedback loop, in which AKT1-driven ROS and oxidized lipids induce CD36, which then amplifies oxLDL uptake and foam cell formation; disruption of AKT1 signaling interrupts this loop. Accordingly, both CD36-blocking peptides and CD36 knockout in *Sr-b1*^*−/−*^*Apoe*^*−/−*^*Cd36*^*−/−*^ mice markedly reduced foam cell formation and coronary atherosclerosis, indicating that CD36 acts as a critical downstream effector of AKT1 in this model [25].

Because of the context-dependent dual role of AKT1 in inflammation and atherogenesis, further investigations are required to clarify its regulatory function within the NF-κB-dependent signaling pathway under different pathophysiological conditions [25].

#### PRKCD

The serine/threonine protein kinase C (PKC) family consists of at least eleven isoforms, which are divided into three subfamilies: classical PKCs (α, βI, βII, γ), novel PKCs (δ, θ, µ, η), and atypical PKCs (ζ ι/λ) [48]. The classical PKCs are diacylglycerol (DAG)- and Calcium (Ca^2+^)-dependent. In contrast, novel PKCs require DAG, but not calcium, for their activation, and atypical PKCs are dependent on neither DAG nor calcium. Still, they are activated by other lipid-based second messengers [24, 49]. When PKCs are stimulated, they anchor to the cell membrane, thereby facilitating their activation and biological effect [49]. Notably, PKCα and PKCβ1 contribute to inflammatory signaling in human monocytes, and their inhibition by the olive oil polyphenol hydroxytyrosol reduces cyclooxygenase-2 (COX-2) and matrix metalloproteinase-9 (MMP-9) expression as well as NF-κB activation [30]. In addition, it was observed that PKCα mediates apelin-13-dependent suppression of lipoprotein lipase (LPL) expression through the apelin receptor (APJ)/PKCα/miR-361-5p signaling pathway in THP-1 macrophage-derived foam cells, thereby reducing lipid accumulation and inflammatory cytokine secretion [31].

It is well established that downregulation of PKCδ reduces oxLDL uptake, accompanied by a concomitant decrease in intracellular lipid accumulation in THP-1-derived macrophages and human primary macrophages [24]. Reduced LDL uptake was observed in THP-1 cells with specific PKCδ short hairpin ribonucleic acid (shRNA) knockdown, as indicated by decreased uptake of fluorescently labeled oxidized and native LDL. Similarly, in primary macrophages treated with the PKCδ inhibitor rottlerin or with PKCδ-specific small interfering RNA (siRNA), both of which reduced oxLDL uptake. These complementary approaches consistently demonstrated that PKCδ regulates the expression of the scavenger receptors SR-A and CD36, both of which were markedly reduced upon suppression of PKCδ [24]. Subsequently, the PI3K/AKT-ERK signaling pathway was identified as a fundamental mechanism underlying SR expression, as targeted blockade of PI3K/AKT with the inhibitor LY294002 and inhibition of ERK with the inhibitor PD98059 in THP-1 cells led to decreased SR-A (both inhibitors) and CD36 (LY294002 only) and reduced p-ERK expression. This effect was confirmed in human primary macrophages treated with LY294002 or *Akt*-targeting siRNA [24].

Furthermore, PKCδ knockdown in THP-1 and human primary macrophages reduced PKCβ protein levels, whereas messenger ribonucleic acid (mRNA) expression remained unchanged [24]. Additionally, ERK inhibition decreased the expression of the PKCβI/βII isoforms. These effects suggest that PKCδ acts upstream of PKCβ in regulating foam cell formation, influencing its protein levels without altering transcription [24].

Under inflammatory and atherosclerotic conditions, increased PKCδ activity, accompanied by elevated AKT and ERK expression, was observed in primary macrophages following stimulation with oxLDL or interferon-gamma (IFN-γ). This effect was confirmed by immunohistochemical (IHC) analysis of human arterial samples from patients undergoing coronary artery bypass grafting (CABG). IHC of these specimens showed increased expression of PKCδ, AKT, p-ERK, and SR-A, whereas CD36 was barely detectable in mildly advanced plaques and in infiltrating macrophages [24].

Based on these results, PKCδ plays a crucial role in foam cell formation and atherosclerosis, underscoring the potential therapeutic value of PKCδ downregulation in reducing atherosclerosis [24].

### CaMK Group

The calcium/calmodulin-dependent protein kinase (CaMK) group comprises serine/threonine kinases, which are regulated by calcium (Ca^2+^) and/or the calcium-binding protein calmodulin (CaM). Therefore, CaMKs mediate Ca^2+^-dependent signaling pathways and regulate processes such as learning and memory, muscle contraction, metabolic regulation, cell proliferation and apoptosis, and gene expression via transcription factors. Additionally, many CaMKs are characterized by an autoinhibitory domain or are activated by autophosphorylation and are Ca^2+^-independent [22, 50].

**Fig. 5:**
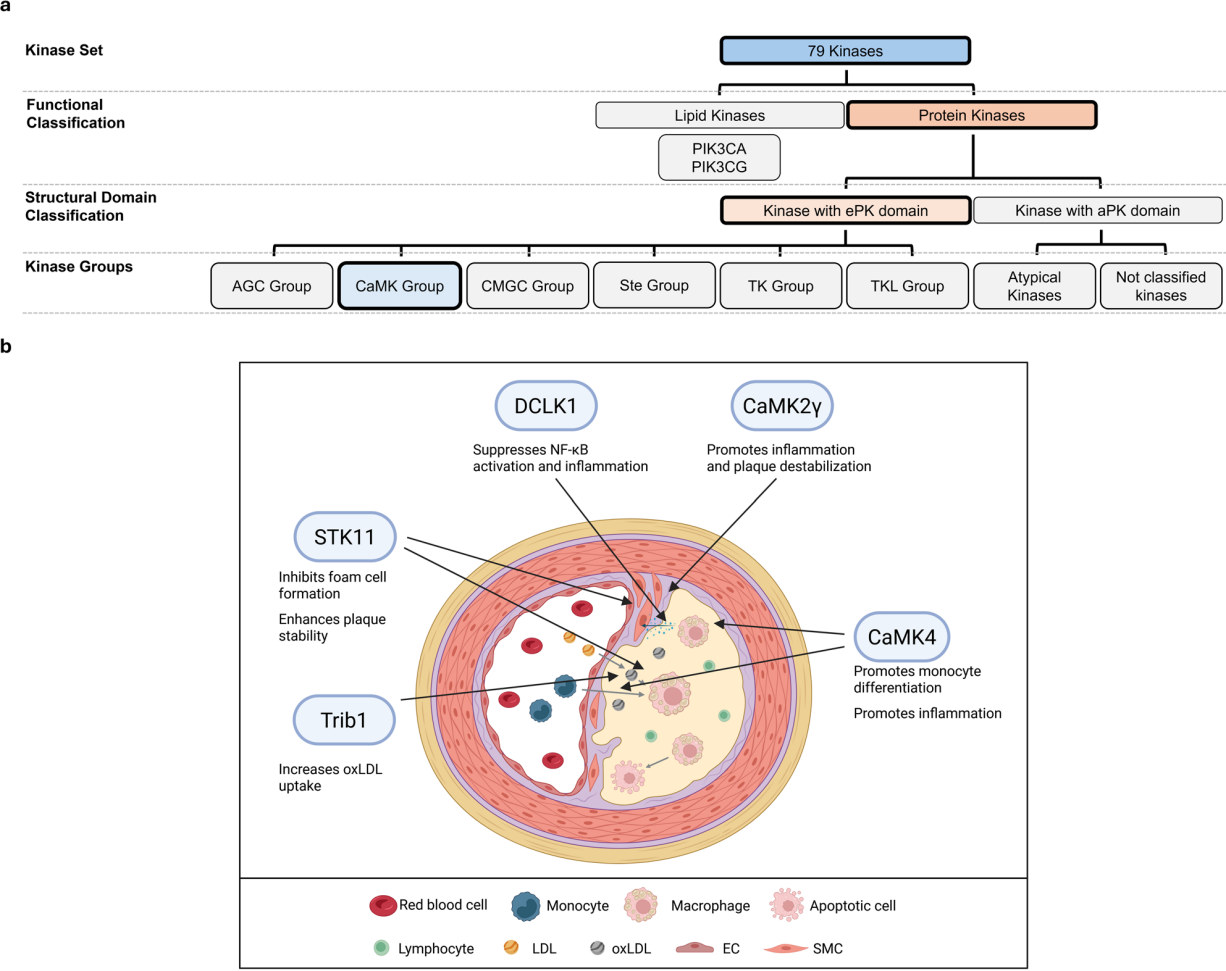
Overview of the role of the CaMK Group of kinases in atherosclerosis. **a** Classification of the 76 kinases into major structural and functional groups, highlighting the CaMK Group. **b** Illustration of the cellular microenvironment of an atherosclerotic lesion and visualizing the involvement of CaMK Group kinases. EC: endothelial cell; LDL: low-density lipoprotein; oxLDL: oxidized LDL; SMC: smooth muscle cell

The CaMKs are already associated with alzheimers disease [51], cardiac hypertrophy and heart failure [52], ischemia [53], schizophrenia [54], and cancer [55, 56]. In atherosclerosis, the kinases CaMK2γ, CaMK4, DCLK1, STK11, and Trib1 have already been described to play an essential role, while additional kinases, such as MAPKAPK2, are relevant in inflammation through monocyte and macrophage activation [57–62] (Fig. 5).

#### CaMK2γ

The calcium/calmodulin-dependent protein kinase type II gamma (CaMK2γ) is active in many tissues and cell types, including neurons, cardiac cells, and immune cells. It regulates cellular processes such as gene expression, ion channel activity, cytoskeletal dynamics, cell survival/death, synaptic plasticity, and immune cell activation [63–66]. Through Ca^2+^ flux, it influences mitochondria (oxidative metabolism, apoptosis), lysosomes (autophagy, Ca^2+^ signaling), the endoplasmic reticulum (ER) (stress response, glucose uptake, tumor metabolism), and modulates voltage-gated Ca^2+^ channels [67–71]. Additionally, it transports Ca^2+/^CaM into the nucleus, phosphorylates the cAMP response element-binding protein (CREB), and thereby regulates the transcription of numerous genes [72]. Besides cancer [73], neurodegenerative diseases [74, 75], and metabolic disorders [76], CaMK2γ also plays a role in CVD [66, 77]. There, its activation in lesional macrophages suppresses the efferocytosis receptor myeloid-epithelial-reproductive tyrosine kinase (MerTK) and promotes plaque necrosis, while nanoparticle-mediated CaMK2γ silencing restores MerTK and stabilizes plaques [61, 78]. Furthermore, CaMK2γ acts as a plaque-destabilizing factor by increasing MMP-9 expression, thereby weakening the fibrous cap. Therefore, the therapeutic relevance of CaMK2γ was investigated by developing a theranostic nanoparticle system (siCaMK2γ-RPP40 NPs). In this system, siRNA nanoparticles were used to specifically inhibit CaMK2γ in macrophages, because it had already been shown that CaMK2γ inhibition led to a simultaneous increase in MerTK expression, a reduction in necrotic core areas, and a thickening of the fibrous cap in *Apoe*^−/−^ mice, thereby improving plaque stability. The nanoparticle system combined targeted siRNA delivery with simultaneous in vivo imaging, which enabled continuous monitoring of therapeutic efficacy and a targeted, locally acting intervention without systemic toxicity. That is why the nanoparticle system was highlighted as a potential therapeutic and diagnostic strategy, which uses a minimally invasive approach, demonstrating that inhibition of the kinase CaMK2γ may represent a promising therapeutic target [61].

#### CaMK4

Calcium/calmodulin-dependent protein kinase IV (CaMK4) is involved in adaptive, general, and inflammatory immunity [79]. It regulates calcium-dependent processes in various cell types, such as macrophages and lymphocytes, and has recently been associated with atherosclerosis [59]. Upstream, calcium/calmodulin-dependent protein kinase kinase 2 (CaMKK2) has been implicated as an important activator of CaMK4 in myeloid cells, suggesting a functional CaMKK2-CaMK4 axis in this context. It was found that CaMK4 is increased in advanced and unstable human carotid artery plaques, as well as in macrophages, and that its loss promotes a pro-reparative phenotype in myeloid cells, suggesting it could be a therapeutic target for atherosclerosis [59].

Studies on *Camk4*^*−/−*^ mice with adeno-associated virus expressing proprotein convertase subtilisin/kexin type 9 (AAV-PCSK9)-induced hypercholesterolemia and a western diet (WD) revealed smaller atherosclerotic plaques, decreased necrotic core area and macrophage content, thicker collagen caps, and no significant differences in body weight, plasma cholesterol, or glucose levels compared with controls [59]. Further analyses of lesional macrophages within plaques of *Camk4*^*−/−*^ mice demonstrated an increase in arginase 1 (Arg1) expression and a higher efferocytosis rate, indicating that macrophages adopt a more anti-inflammatory and reparative phenotype in the absence of CaMK4. In addition, the loss of *Camk4* altered circulating leukocyte profiles, with elevated numbers of lymphocyte antigen 6 complex, locus C (Ly6C)^low^ monocytes and unchanged Ly6C^high^ monocytes, confirming that CaMK4 influences monocyte differentiation and promotes a shift toward the anti-inflammatory subset [59]. Consistently, human peripheral blood mononuclear cells (PBMCs) treated with the CaMK inhibitor N-[2-[[[3-(4-Chlorophenyl)-2-propenyl]methylamino]methyl]phenyl]-N-(2-hydroxyethyl)-4-methoxybenzenesulfonamide (KN93) or transfected with *Camk4*-targeting siRNA displayed the promotion of non-classical CD14^−^CD16^+^ monocyte differentiation instead of the classical CD14^+^CD16^−^ monocytes, demonstrating a role for CaMK4 in human monocyte differentiation [59]. Additional effects of *Camk4* loss were observed in the bone marrow by flow cytometry and the MethoCult assay, which showed an increase in total myeloid colonies compared with marrow isolated from control mice [59]. Mechanistically, *Camk4* deficiency was associated with altered activating transcription factor 6 (ATF6)-dependent transcription, as *Camk4*^*−/−*^ monocytes showed increased *Atf6* and decreased *Atf3* expression, both of which favor non-classical monocyte differentiation. Chromatin immunoprecipitation (ChIP) analyses further confirmed stronger *Atf6* binding to the nuclear receptor subfamily 4, group A, member 1 (*Nr4a1*) enhancer, consistent with *Nr4a1*’s role as a key transcriptional regulator of the Ly6^low^ monocyte subset. Together, these findings indicate that CaMK4 modulates monocyte lineage commitment through an ATF6-NR4A1-dependent pathway [59].

*Camk4* ^*−/−*^ monocyte migration was also altered, with lower C-C chemokine receptor type 2 (*Ccr2*) and higher *Ccr5* expression, resulting in less efficient migration toward the CCR2 ligand C-C motif chemokine ligand 2 (CCL2), but enhanced migration toward CCR5 ligands [59]. In vivo bead-tracking assays confirmed that fewer *Camk4*^*−/−*^ monocytes entered the spleen and atherosclerotic plaques, indicating reduced recruitment to inflamed tissues [59]. The loss of *Camk4* also affected macrophage polarization and function. *Camk4*^*−/−*^ macrophages expressed higher levels of Arg1, IL-10, C-X3-C motif chemokine receptor 1 (CX3CR1), and NR4A1, but lower levels of CCR2, and exhibited increased efferocytosis along with elevated secretion of the pro-resolving mediators lipoxin A4 and resolving D1 (RvD1). The absence of CaMK4, therefore, promotes resolution and reduces inflammation. In line with this, human monocyte-derived macrophages (MDMs) treated with KN93 or a *Camk4*-targeting siRNA showed reduced expression of IL-1β, IL-6, and inducible nitric oxide synthase (iNOS) (M1 markers). At the same time, they increased Arg1 and IL-10 (M2 markers), confirming that inhibition of CaMK4 favors anti-inflammatory polarization in human cells [59]. Therefore, CaMK4 serves as a molecular marker of advanced atherosclerosis and may be a potential therapeutic target for further investigation [59].

#### DCLK1

Doublecortin-like kinase protein 1 (DCLK1) is a serine/threonine kinase associated with microtubules. It was initially described in the nervous system, where it is involved in both the regulation of microtubule polymerization [80] and the control of neuronal migration [81]. In addition to the nervous system, DCLK1 is expressed primarily in the gastrointestinal tract, where it marks post-mitotic tuft cells that function as sensory or niche-regulating cell types [82, 83]. Although DCLK1-positive cells are not classic stem cells, they play an essential role in tissue homeostasis and regeneration [84]. Based on existing knowledge of DCLK1’s role in tumor biology, particularly in inflammatory processes, it has been investigated whether DCLK1 is associated with atherosclerosis [58].

In *Apoe*^−/−^ mice, DCLK1 inhibition or deletion reduced aortic inflammation and prevented inflammatory cell infiltration. In an in vitro model of oxLDL-stimulated primary macrophages, inhibition using the pharmacological DCLK1-inhibitor-1 (DCLK1-IN-1) or deletion of DCLK1 significantly reduced the expression and secretion of pro-inflammatory cytokines, including TNF and IL-6, indicating suppression of NF-κB-mediated macrophage activation. In contrast, in the absence of *Dclk1* inhibition or knockout, elevated DCLK1 levels in macrophages promoted oxLDL-induced inflammation and exacerbated atherosclerosis in vivo [58]. Using Western blotting and co-immunoprecipitation, phosphorylation of IKKβ at Ser177/181 by DCLK1 was demonstrated, and DCLK1-mediated abolition of NF-κB activation was achieved by IKKβ knockdown, showing a strong interaction between DCLK1 and NF-κB-dependent signaling [58]. Thereby, this study identified DCLK1 as a potential therapeutic target for attenuating atherosclerosis.

#### STK11

The constitutively active master serine/threonine kinase 11 (STK11, also known as liver kinase B1, LKB1) regulates cellular metabolism and polarity through a complex signaling network. Together with the pseudo-kinase STE20-related adaptor (STRAD) and the scaffolding protein calcium-binding protein 39 (CAB39), STK11 forms a heterotrimeric complex [85, 86] that leads to its translocation from the nucleus to the cytoplasm. There, it phosphorylates adenosine monophosphate-activated protein kinase (AMPK) and 12 other AMPK-related kinases [87–89]. In addition to its pleiotropic role in cancer biology [89], STK11 has also been shown to be relevant in atherosclerosis [60]. In atherosclerotic plaques from patients and in human oxLDL-stimulated macrophages, STK11 was reduced, and its activity inhibited foam cell formation by regulating lipid uptake and accumulation. Moreover, the itchy E3 ubiquitin protein ligase (ITCH) promotes STK11 degradation, mediated by the long non-coding RNA nuclear-enriched abundant transcript 1 (NEAT1), which acts as a sponge for microRNA (miR)-17-5p, thereby facilitating atherosclerosis development. Consistent with this, STK11 has a protective role in atherosclerosis [60]. A mechanistic link between STK11 and plaque instability was also demonstrated, which showed that the advanced glycation end product Nε-carboxyethyl-lysine (CEL) impairs macrophage autophagy and promotes plaque instability by suppressing the receptor for advanced glycation end products (RAGE)/STK11/AMPK/Sirtuin 1 (SIRT1) signaling axis [90]. In this pathway, CEL binding to RAGE reduces STK11 and AMPK phosphorylation and decreases SIRT1 expression and catalytic activity. This leads to impaired autophagy through the acetylation and nuclear translocation of zinc finger with Krüppel-associated box and SRE-zinc finger domain-associated domain 3 (ZKSCAN3), thereby destabilizing atherosclerotic plaques, particularly under diabetic conditions [90].

#### Trib1

Tribbles 1 (Trib1) is a serine-threonine kinase-like protein that is ubiquitously expressed, mainly in the liver [91] and coronary arteries [57]. Its expression is particularly elevated in advanced coronary heart disease [92] and has been detected in murine plaque macrophages [93]. Although the exact functions of Trib1 are not yet fully understood, it is assumed to act as an adaptor protein in various signaling pathways [91]. Liver-specific *Trib1*-deficient mice show elevated plasma triglyceride and cholesterol levels [94], while hematopoietic *Trib1*-deficiency causes a decreased presence of M2-like (F4/80^+^MR^+^) macrophages in various murine organs [95]. Overexpression, by contrast, leads to normalization of lipid parameters [96]. Trib1 has therefore already been associated with coronary heart disease and with dyslipidemia [57, 97].

More detailed investigations into the functions of Trib1 in macrophages during atherosclerosis demonstrated that a myeloid-specific *Trib1* (*mTrib1*) deficiency in *Apoe*^*−/−*^ mice reduced early atheroma formation, whereas *mTrib1* overexpression enhanced atherogenesis [57]. The manipulation of *Trib1* expression was restricted to the myeloid lineage, primarily affecting macrophages and, to a lesser extent, neutrophils. Mechanistically, *mTrib1* overexpression increased oxidized low-density lipoprotein receptor 1 (Olr1) expression in macrophages, thereby enhancing oxLDL uptake, increasing lipid accumulation, and expanding foam cell numbers within the plaque, while circulating lipid levels remained unchanged [57]. Subsequently, a second model, using adeno-associated virus serotype 8 expressing PCSK9 (AAV8-PCSK9) in mTrib1 overexpression mice, confirmed that this effect was independent of plasma lipid levels [57]. Histological analysis revealed that *Trib1* overexpression increased the number and size of foam cells. At the same time, *Trib1* deficiency reduced them, indicating that Trib1 acts through foam cell expansion and enhanced lipid uptake rather than macrophage polarization [57]. To link these findings to humans, data from the Cardiogenics Transcriptomics Study [98] were analyzed to compare Trib1^high^ and Trib1^low^ macrophages. Expression of OLR1, the receptor for oxLDL, was markedly increased in Trib1^high^ cells. In contrast, other lipid transport genes (LDL receptor (LDLR), SRB1, ATP-binding cassette transporter A1 (ABCA1), ABCG1) were less affected, suggesting a specific connection between Trib1 and OLR1 [57]. This link was confirmed in BMDMs from *Trib1*-overexpressing mice and in wild-type mice, demonstrating elevated Olr1 expression, reduced Srb1 expression, and more Olr1-positive cells in Trib1-overexpressing macrophages. Moreover, BMDMs from *Trib1*-overexpressing mice accumulated higher amounts of cholesterol. They formed nearly three times as many foam cells after oxLDL exposure, while high-density lipoprotein (HDL)-mediated cholesterol efflux was unchanged [57].

In summary, Trib1 in myeloid cells promotes atherosclerosis by upregulating Olr1, increasing oxLDL uptake, and driving foam cell formation, whereas *Trib1* deficiency is protective. These results suggest that selective inhibition of Trib1 in macrophages could help limit atherogenesis beyond conventional lipid-lowering therapy [57].

### CMGC Group

The CDK, MAPK, GSK, and CDK-like related group (CMGC) comprises cyclin-dependent kinases (CDKs), mitogen-activated protein kinases (MAPKs), glycogen synthase kinases (GSKs), and CDK-like kinases (CLKs). Their functions include regulating the cell cycle, mediating signal transduction via MAPK cascades, modulating gene expression, and supporting proliferation, differentiation, apoptosis, and metabolism. Therefore, CMGC kinases are key regulators of eukaryotic signaling networks and can lead to cancer, metabolic disorders, and neurodegenerative or CVD when dysregulated [22, 99].

**Fig. 6:**
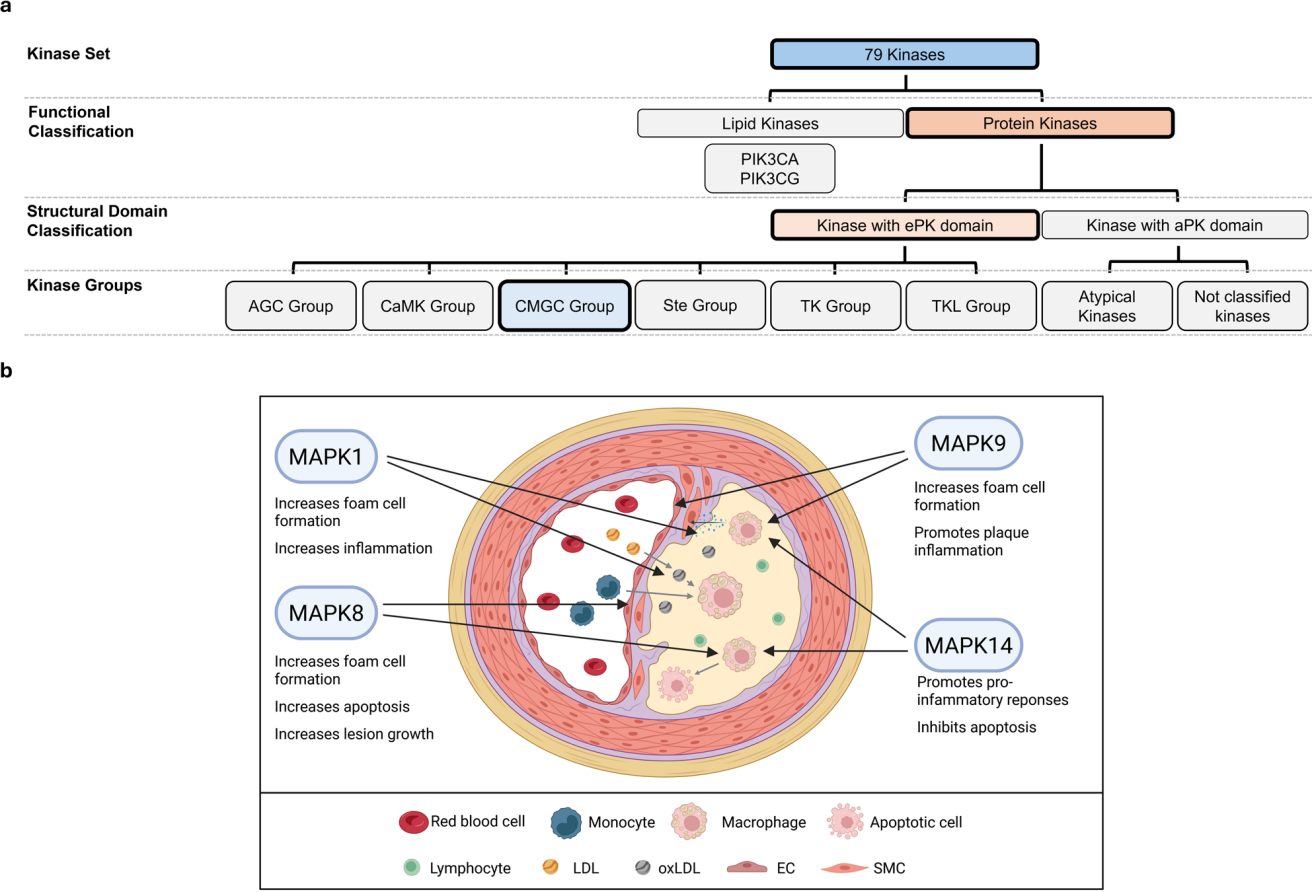
Overview of the role of the CMGC Group of kinases in atherosclerosis. **a** Classification of the 76 kinases into major structural and functional groups, highlighting the CMGC Group. **b** Illustration of the cellular microenvironment of an atherosclerotic lesion and visualizing the involvement of CMGC Group kinases. EC: endothelial cell; LDL: low-density lipoprotein; oxLDL: oxidized LDL; SMC: smooth muscle cell

The kinases MAPK1, MAPK14, MAPK8 (JNK1), and MAPK9 (JNK2) have already been investigated for their roles in atherosclerosis [100–106]. In contrast, the contributions of other kinases, such as CDK4, GSK3B, and MAPK7, remain less well characterized but have been reported in relation to atherosclerosis [107, 108] (Fig. 6).

#### MAPK1

The MAPK1, also called ERK2, acts through the rat sarcoma virus-related small GTPase (Ras)-dependent rapidly accelerated fibrosarcoma kinase (Raf)-MAPK/ERK kinase (MEK)-MAPK cascade. ERK2 is only activated by the dual activity of the upstream MEK1/2 kinase. MEK1/2 phosphorylates ERK1/2 at its threonine and tyrosine residues within its activation loop (threonine-glutamate-tyrosine (TEY) motif) [109]. After activation, ERK2 can influence various cellular processes, including embryogenesis, cell proliferation and differentiation, and apoptosis. When ERK2 is overexpressed or constitutively activated, it promotes the progression of multiple cancers [110]. Studies have shown that different types of inhibitors can reduce inflammation, as demonstrated for MEK1/2 inhibitors, which can specifically block ERK1/2 activity due to their high substrate specificity [103, 111, 112]. Similarly, antioxidant plant compounds such as the lignan epsisamin, have been shown to suppress ERK2 activation and downstream NF-κB-dependent signaling [113, 114].

Furthermore, the combination of a MEK1/2 inhibitor (U0126) and a liver X receptor (LXR) ligand (T0901317) was tested in an *Apoe*^*−/−*^ mouse model and human cells (human epithelioma G2 (HepG2), human umbilical vein endothelial cells (HUVEC), SMCs) to investigate a possible synergistic effect in reducing atherosclerosis [103]. The mouse models were primarily treated with the MEK1/2 inhibitor, either alone or in combination with the LXR ligand. MEK1/2 inhibition in vitro with the inhibitor PD98059 markedly reduced hepatic lipid accumulation, hypertriglyceridemia, and liver damage, while enhancing anti-atherogenic effects. Combining with the LXR ligand further amplified these beneficial effects and significantly reduced its adverse metabolic effects [103]. MEK1/2 inhibition also promoted ABCA1 expression and cholesterol efflux in macrophages, thereby reducing foam cell formation. In combination with the LXR ligand, these effects were synergistically enhanced, resulting in a strong induction of reverse cholesterol transport [103]. Although the combined treatment did not affect serum LDL or HDL cholesterol levels, it maintained arterial wall integrity, reduced macrophage accumulation in lesions and VCAM-1 expression, thereby reducing monocyte adhesion and foam cell formation. Consequently, atherosclerotic lesions in *Apoe*^*−/−*^ mice were markedly reduced. In addition, total and very low-density lipoprotein (VLDL) cholesterol levels were lowered, and LDL cholesterol was reduced in a regression model using a regular diet [103]. Furthermore, inhibition of ERK1/2 with the MEK1/2 inhibitor U0126 prevented the lipogenic and hepatotoxic effects typically induced by the LXR ligand T0901317. These findings indicate a potential new therapeutic approach for reducing atherosclerosis with a lower risk of metabolic side effects [103].

In addition to the described lipid metabolic effects, ERK1/2 also regulates inflammatory processes. Meprin-β-induced ERK1/2 phosphorylation via A disintegrin and metalloproteinase 10 (ADAM10) led to NF-κB activation and the production of pro-inflammatory cytokines, including IL-1β, IL-6, and IL-18, in macrophages. Inhibition of ERK1/2 with PD98059 suppressed this response by blocking NF-κB activation, demonstrating an additional anti-inflammatory effect of MEK/ERK pathway inhibition [104].

#### MAPK8/MAPK9/MAPK10

The c-Jun N-terminal kinases (JNKs), also known as MAPK8 (JNK1), MAPK9 (JNK2) and MAPK10 (JNK3) are stress-activated serine/threonine protein kinases of the MAPK family [115, 116]. Typical activating factors include environmental stress [117], pro-inflammatory cytokines (TNF, IL-1) [118], and TLR ligands [119]. While JNK1/2 are ubiquitously expressed, JNK3 is largely restricted to the brain, heart, and testis [116]. Their isoform-specific functions are increasingly recognized, whereas they were initially thought to be largely isoform-independent [120]. JNKs are activated through sequential activation of dual-specificity MAP kinase kinases (MKK), MKK4, and MKK7, and MAP kinase kinase kinases (MKKK), including apoptosis signal-regulating kinase 1 (ASK1), MAPK/ERK kinase kinase (MEKK1-4), transforming growth factor-β-activated kinase (TAK1-3), or mixed-lineage kinase (MLK1-3), depending on cell lineage and stimulus [117, 121]. After activation, JNKs modulate gene expression programs that control proliferation [122], apoptosis [123], migration [124], angiogenesis [125], and inflammatory responses [119]. Their role has been identified in various inflammatory diseases, including rheumatoid arthritis [126], psoriatic arthritis [127], and systemic lupus erythematosus [128], and, more recently, in atherosclerosis, where JNK is implicated in vascular inflammation and lipid metabolism [115, 129].

The investigation of JNK’s molecular function and that of its isoforms includes their putative roles in ECs and macrophages, encompassing apoptosis, oxidative stress, foam cell formation, and vascular barrier regulation. Therefore, various strategies encompassing nonspecific inhibitors targeting all isoforms (CT536706, SP600125), JNK2/3-selective inhibitors (JNK inhibitor IX), knockdown with JNK1- and JNK2-siRNA, p5RHH-JNK2-siRNA-nanoparticles, or *Jnk1*^*−/−*^ and *Jnk2*^*−/−*^ mouse models have been performed [100–102, 105, 106].

JNK1 and JNK2 were for example investigated in hematopoietic cells, particularly in macrophages that predominate in early lesions, in *Ldlr*^*−/−*^ bone marrow chimeras, demonstrating increased lesions, more lesional macrophages, and less apoptosis in *Ldlr*^*−/−*^ mice reconstituted with *Jnk1*^*−/−*^ bone marrow, whereas *Ldlr*^*−/−*^ mice reconstituted with *Jnk2*^*−/−*^ bone marrow showed no relevant changes [101]. Reduced total JNK activity by knockdown to a single functional allele in vivo (*Jnk1*^*+/−*^*/Jnk2*^*−/−*^ or *Jnk1*^*−/−*^*/Jnk2*^*+/−*^) in hematopoietic cells resulted in even larger lesions than before, indicating that the extent of JNK signaling in hematopoietic cells scales the atherosclerotic burden. Further in vitro analyses on peritoneal macrophages revealed JNK1’s crucial interaction with the AKT signaling pathway, while JNK2 had no significant role. Because JNK1 downregulates AKT, the significance for cell fate was clarified by showing that palmitate-induced ER stress progressively increased TUNEL-positive apoptosis in WT macrophages, while *Jnk1*^*−/−*^ macrophages were largely protected from ER-stress-induced cell death. Building on this, PTEN was identified as the critical link between JNK and AKT, showing that in *Pten*^*−/−*^ macrophages JNK activation was no longer able to suppress AKT, indicating that PTEN mediates the JNK-dependent inhibition of AKT and thus couples stress-activated JNK1 signaling to diminished AKT survival signaling. Based on these findings, JNK1 may be a potential therapeutic target in cardiovascular metabolic diseases, but targeted, cell-type-specific, and stage-dependent modulation may be essential, as JNK1 deficiency reduced apoptosis but promoted early atherosclerosis [101]. In line this study, BMDMs from C57BL/6 mice were used to dissect LPS-induced foam cell formation, revealing, by pharmacologic JNK inhibition and isoform-specific knockdown with JNK1- or JNK2-siRNA, that a JNK1-dependent CD14/SR-AI signaling pathway is the key upstream mediator of LPS-induced CD14/SR-AI expression, oxLDL uptake, and macrophage foam cell formation [130].

Although JNK2 showed no relevant effects on atherosclerosis in the described JNK1 studies, JNK2-specific studies point out its proatherogenic actions, particularly by promoting SR-dependent foam cell formation and contributing to hypercholesterolemia-induced endothelial dysfunction, oxidative stress, and plaque inflammation [105, 106].The aortas of C57BL/6J mice with hypercholesterolemia showed increased JNK phosphorylation compared with WT on a normal diet, indicating that hypercholesterolemia induces aortic JNK activation. Subsequent *Jnk2*^*−/−*^ knockout mouse studies demonstrated no endothelial dysfunction and preserved acetylcholine-induced relaxations, whereas WT mice on hypercholesterolemia showed opposite effects. Furthermore, polyethylene glycol-superoxide dismutase (PEG-SOD) treatment revealed that scavenging superoxide markedly improved endothelium-dependent relaxation in hypercholesterolemic WT mice, underscoring a JNK2-linked, ROS-mediated mechanism of endothelial dysfunction. Therefore, JNK2 deletion protects against hypercholesterolemia-induced endothelial dysfunction, and the improvement by PEG-SOD suggests an important role of reactive oxygen species in the dysfunction observed in WT mice on HCD [105]. Building on these insights from genetic JNK2 deletion in hypercholesterolemic mice, subsequent work shifted from germline knockout models to targeted JNK2 inhibition in established plaques, using peptide-siRNA nanostructures (p5RHH-JNK2 siRNA) to locally silence JNK2 in plaque macrophages of *Apoe*^*−/−*^ mice on a WD. This strategy revealed that JNK2 silencing in advanced atherosclerosis reduces macrophage burden, inflammatory signaling (NF-κB, STAT3), endothelial barrier disruption, and thrombotic risk [106]. In vitro analyses of RAW 264.7 macrophages treated with p5RHH-JNK2 siRNA nanoparticles demonstrated substantially reduced cellular uptake of acLDL and markedly diminished lipid accumulation compared with untreated controls. In *Apoe*^*−/−*^ mice, short-term treatment with p5RHH-JNK2 siRNA nanoparticles significantly attenuated thrombotic risk, restored endothelial barrier integrity in atherosclerotic plaques, and reduced both plaque necrotic area and macrophage burden [106]. Based on these findings, targeted inhibition of JNK2 with p5RHH-JNK2 siRNA nanoparticles may reduce atherothrombotic risk and promote plaque healing without causing relevant systemic immune side effects [106].

#### MAPK14

The kinase MAPK14 (p38α) belongs to the MAPK family [131]. It is activated by a broad range of environmental and inflammatory stimuli, including oxidative and osmotic stress, cytokines, bacterial components, UV exposure, and low oxygen levels [132]. Among the four p38 MAPK isoforms, MAPK14 is the most abundantly expressed and plays a central regulatory role in processes including cell growth, differentiation, apoptosis, inflammation, and immune regulation [133]. One cellular function of MAPK14 is the regulation of cytokine production and gene expression, as nearly half of its substrates are transcription factors [133–135]. Due to its broad regulatory effects on cellular stress and signaling pathways, MAPK14 has been linked to cancer, neurodegenerative diseases, inflammatory diseases, and CVD [136].

The role of MAPK14 in atherogenesis, particularly under the influence of enzymatically modified low-density lipoproteins (eLDL), was investigated. eLDL represents a form that is enzymatically modified by hydrolytic enzymes such as trypsin and cholesterol esterase, leading to proteolytic and lipolytic alterations of the LDL particle. Unlike oxLDL, this modification is non-oxidative and results from enzymatic degradation within the arterial intima [137]. MAPK14 was identified as the dominant isoform of the p38-MAPK family that colocalizes with eLDL in atherosclerotic lesions of carotid specimens. In functional experiments using THP-1 cells and primary monocyte-macrophages, inhibition of MAPK14 with the selective inhibitor Skepinone-L markedly reduced eLDL-induced activation of the p38 signaling pathway [137]. Additionally, the expression of lipid transport proteins CD36 and ABCA1 was reduced, without affecting foam cell formation. Furthermore, inhibition of MAPK14 produced distinct effects on apoptosis in THP-1 cells and primary monocyte-macrophages. While no effect on eLDL-induced apoptosis could be observed in THP-1 cells, there was significant inhibition of caspase-3/7 activity (reduced apoptosis) in primary monocyte-macrophages. Additionally, the secretion of pro-inflammatory cytokines was significantly inhibited [137]. Thus, MAPK14 plays a central role in inflammatory processes during atherogenesis, making selective inhibition a potential therapeutic approach to modulate pathological inflammatory cascades [137].

### Ste Group

The sterile (Ste) kinase group comprises three primary MAPK cascade families: Ste7/MAP2K, Ste11/MAP3K, and Ste20/MAP4K. The MAPK cascade is conserved in eukaryotes and begins with Ste11 phosphorylation by Ste20; Ste11 then phosphorylates Ste7, which subsequently phosphorylates MAPK. Small G proteins primarily initiate this signaling cascade by sequentially activating kinases. Overall, this system coregulates different cellular and (patho)physiological processes and is a central part of eukaryotic signaling networks [22, 99].

**Fig. 7:**
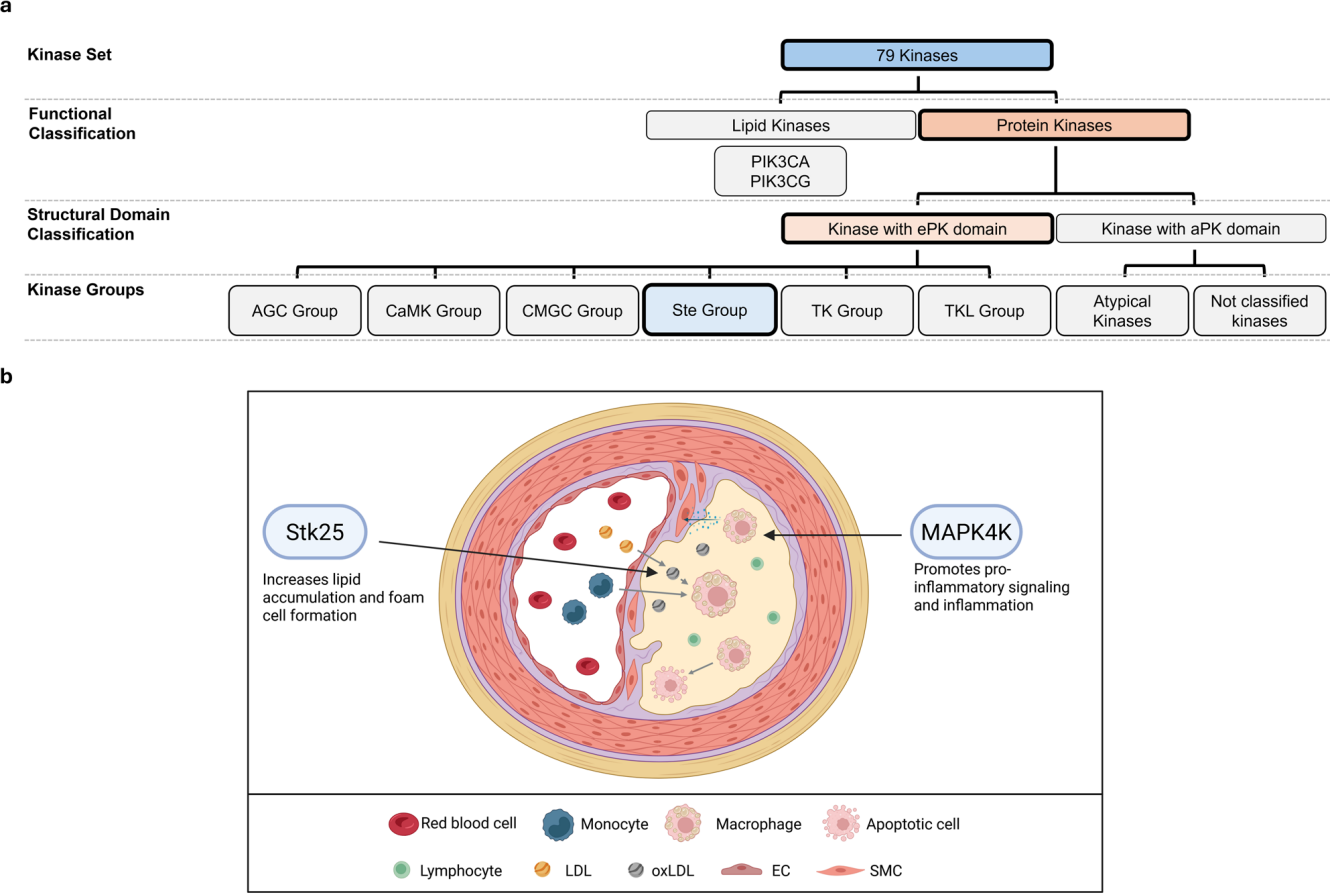
Overview of the role of the Ste Group of kinases in atherosclerosis. **a** Classification of the 76 kinases into major structural and functional groups, highlighting the Ste Group. **b** Illustration of the cellular microenvironment of an atherosclerotic lesion and visualizing the involvement of Ste Group kinases. EC: endothelial cell; LDL: low-density lipoprotein; oxLDL: oxidized LDL; SMC: smooth muscle cell

In atherosclerosis, MAP4K4 (Ste20 family) and serine/threonine kinase 25 (Stk25) (Ste20 family) have been examined in more detail already, whereas other kinases, such as MAP2K1 (Ste7 family), MAP3K5 (Ste11 family), MAP3K7 (Ste11 family), and TNF receptor-associated factor 2 (Traf2) and non-catalytic region of tyrosine kinase adaptor protein (Nck)-interacting kinase (TNIK) (Ste20 family), have been associated with the disease but not explicitly characterized in terms of their roles in its pathogenesis [22, 138–143] (Fig. 7).

#### MAP4K4

The serine/threonine kinase MAP4K4 belongs to the Germinal Cell Kinase Four (GCK-IV) group and is part of the Ste20/MAP4K family [144]. It is broadly expressed in various tissues and is mainly involved in cellular processes such as cytoskeletal dynamics, ion transport, cell proliferation, and survival [145–151]. This kinase has previously been associated with inflammation and atherosclerosis, suggesting a promising therapeutic target for a selective small-molecule inhibitor of MAP4K4 [138, 152]. MAP4K4 was identified as a pro-inflammatory gene regulating expression in macrophages using β1,3-d-glucan-encapsulated siRNA particles (GeRPs). This effective oral delivery system silences genes in mouse macrophages both in vitro and in vivo [138]. As a result of targeted gene silencing of MAP4K4 in peritoneal macrophages, its mRNA expression was reduced by approximately 70–80%. Additional stimulation with lipopolysaccharide (LPS) in peritoneal exudate cells significantly inhibited the inflammatory response in macrophages, with approximately 50% reduced *Tnf* expression and 30% lower TNF protein levels in the medium. Furthermore, 5’ rapid amplification of cDNA ends (RACE) analysis confirmed that the observed *Map4k4* silencing occurred through an siRNA-mediated RNA interference (RNAi) mechanism. Additionally, Western blot analysis of the known inflammatory signaling pathways JNK, p38, ERK, and NF-κB after LPS stimulation and *Map4k4* silencing in macrophages showed no changes in their activation, indicating that MAP4K4 mediates a pro-inflammatory signaling pathway independent of the classical MAPK and NF-κB cascades [138].

Because of these findings and the strong protective effect without toxicity observed in mouse models at just 20 µg siRNA/kg, the GeRP method was identified as an efficient and safe approach for systemic siRNA delivery to macrophages in vivo. Furthermore, the findings on *Map4k4* silencing suggest that it may be a therapeutic target for inflammatory diseases, particularly atherosclerosis [138].

#### Stk25

The serine/threonine protein kinase 25 (Stk25) is part of the GCKIII subgroup of MST protein kinases (Sterile20-like kinases) within the GCK family. It is a critical regulator of ectopic lipid storage, glucose and insulin homeostasis, fibrosis, and meta-inflammation [153, 154]. Primarily, Stk25 is involved in cellular processes such as autophagy, cell polarity, apoptosis, and cell migration, and plays a role in cardiovascular development [154–160]. Furthermore, Stk25 influences lipid oxidation, glucose tolerance, and insulin sensitivity, which is why it is already considered a therapeutic target for metabolic diseases such as type 2 diabetes [161–164]. Furthermore, Stk25 was detected in all aortic layers, suggesting a direct role in CVD [164]. The effects of Stk25 overexpression were assessed in mice with atherosclerosis induced by AAV8-hPCSK9 gene transfer combined with an atherogenic WD for 12 weeks. Hepatic LDLR abundance was markedly reduced, whereas atherosclerotic plaques were larger, with increased collagen deposition and oxidative stress, and increased macrophage infiltration from the circulation [164]. In parallel, triacylglycerol accumulation in the liver increased, accompanied by pronounced macro- and microvesicular steatosis, increased macrophage infiltration, and more severe fibrosis, supporting a link between fatty liver pathology and vascular changes [164]. In line with this, deletion of Stk25 in the same atherosclerosis model resulted in smaller atherosclerotic lesions, reduced macrophage infiltration, decreased collagen content, and a trend toward lower oxidative stress. At the same time, hepatic burden was reduced, with less triacylglycerol accumulation and attenuated fibrosis [164]. Collectively, these findings indicate that loss of *Stk25* can protect against atherosclerosis and highlight Stk25 as a potential therapeutic target for preventing atherosclerotic disease [164].

### TK Group

The tyrosine kinase (TK) group encompasses enzymes that catalyze the transfer of phosphate groups to specific tyrosine residues within substrate proteins, including cytoplasmic targets and the intracellular domains of transmembrane receptors. This phosphorylation mechanism modulates intracellular signaling pathways by altering the enzymatic activity of target proteins or by generating binding sites for downstream signaling molecules such as adaptor proteins. Consequently, TKs exert a crucial regulatory influence on cell adhesion, motility, proliferation, cell cycle progression, and apoptosis, as well as on organismal processes including growth, development, metabolism, and immune defense. Dysregulated TK-mediated signaling represents a common oncogenic mechanism, linking TKs to tumor initiation and progression. Their aberrant activity can be therapeutically inhibited by tyrosine kinase inhibitors (TKIs) such as imatinib, gefitinib, erlotinib, or sunitinib [165].

**Fig. 8:**
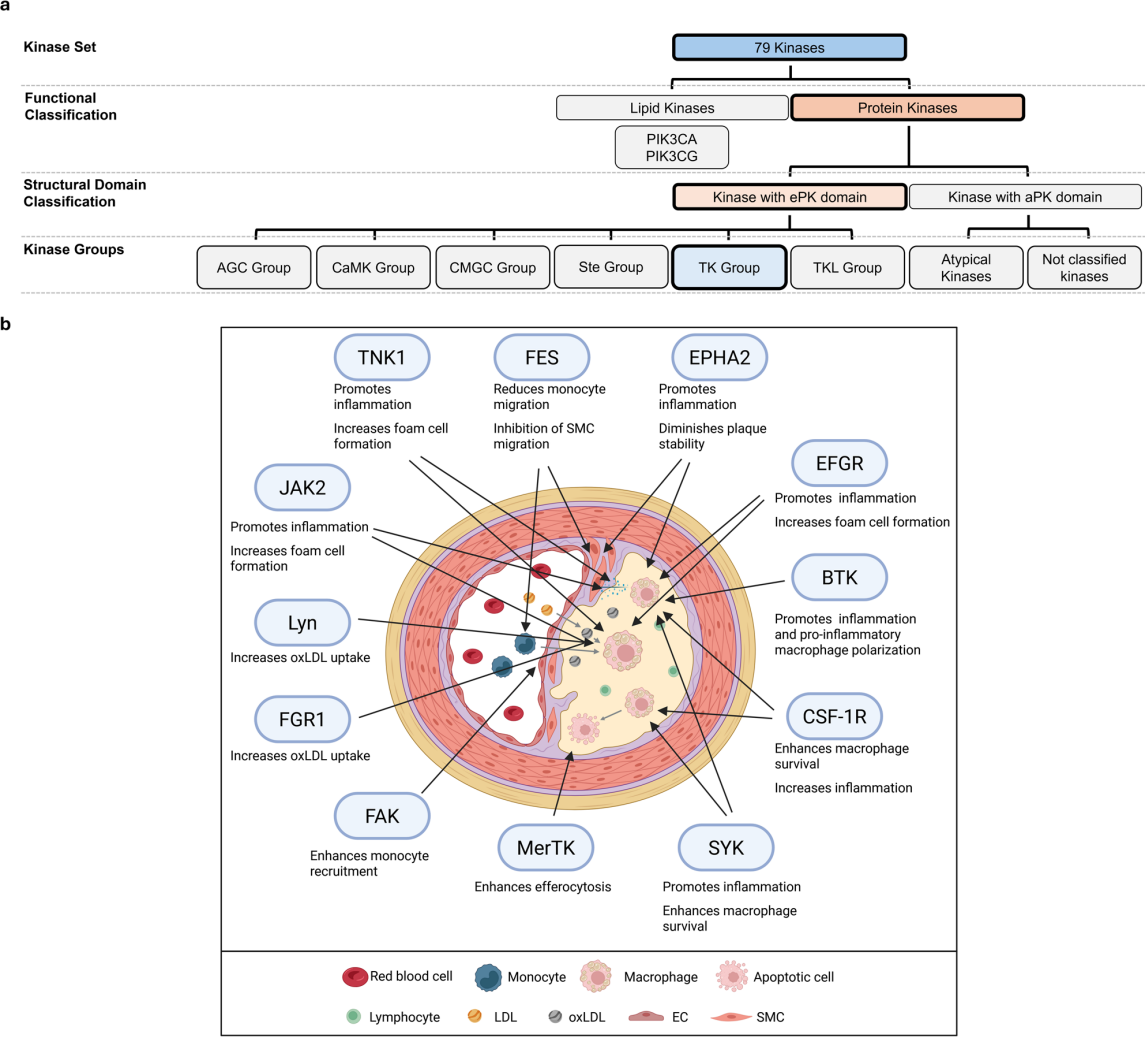
Overview of the role of the TK Group of kinases in atherosclerosis. **a** Classification of the 76 kinases into major structural and functional groups, highlighting the TK Group. **b** Illustration of the cellular microenvironment of an atherosclerotic lesion and visualizing the involvement of TK Group kinases. EC: endothelial cell; LDL: low-density lipoprotein; oxLDL: oxidized LDL; SMC: smooth muscle cell

Several TK kinases were further investigated in their role in atherosclerosis. These include BTK, CSF-1R, EGFR, EphA2, FES, FGFR1, JAK1, JAK2, JAK3, Lyn, MerTK, FAK, PYK2, SYK, and TNK1 [166–181]. Other kinases, such as HCK, IGF1R, TEK, TYK2, FLT1, FLT3, KDR, and SRC, are associated with atherosclerosis, but their exact mechanistic roles remain rather elusive [182–195] (Fig. 8).

#### BTK

The non-receptor-associated Bruton’s tyrosine kinase (BTK) is involved in cell development, proliferation, and differentiation, and is part of innate and adaptive immunity [196, 197]. Specifically in macrophages, BTK is associated with polarization, phagocytosis, and pro-inflammatory secretion [198, 199], which is why BTK was identified as a shared immune-related hub gene in atherosclerosis [166, 200]. Furthermore, it is already a well-established target in autoimmune diseases and B-cell malignancies [166, 167, 201–203].

Investigation of the mechanistic role of BTK in macrophage-mediated atherosclerosis revealed a correlation between BTK and increased oxidative stress, ER stress, and inflammation. BTK’s influence on atherosclerosis was further confirmed by oxLDL-treated RAW264.7 macrophages and human aortic plaques, which both showed higher BTK expression levels. In contrast, BTK-targeted siRNA reduced the secretion of the pro-inflammatory cytokines TNF and IL-6, and shifted macrophage polarization toward the anti-inflammatory M2 phenotype [166]. Macrophages treated with BTK-siRNA and oxLDL exhibited reduced oxidative and ER stress. This was accompanied by enhanced nuclear factor erythroid 2-related factor 2 (NRF2) activation and decreased expression of the mitochondrial fission factors dynamin-related protein 1 (DRP1) and fission 1 protein (FIS1), indicating diminished mitochondrial damage. Moreover, nuclear translocation of ATF6 was inhibited, reducing phosphorylation of protein kinase R-like ER kinase (PERK) and inositol-requiring enzyme 1 (IRE1) and thereby alleviating ER stress [166]. These findings were confirmed in an *in vivo Apoe*^*−/−*^ mouse model fed a high-fat diet (HFD) and treated with an injection of a sh-BTK adenovirus. These mice showed reduced plaque formation, smaller necrotic cores, and fewer foam cells, suggesting that BTK is a potential therapeutic target for atherosclerosis [166].

Further investigations focused on developing a CD47-targeted polydopamine-based nanocarrier system (PIP-CD47) to deliver the BTK inhibitor ibrutinib to atherosclerotic plaques. BTK was successfully inhibited in human macrophages treated with PIP-CD47, leading to suppressed nucleotide-binding oligomerization domain (NOD)-like receptor family, NLRP3 inflammasome activity, decreased IL-1β secretion, and a shift towards M2 polarization, thereby stabilizing plaques, reducing inflammation, oxidative stress, and foam cell formation. Furthermore, BTK inhibition in B cells suppresses BCR-NF-κB-Bcl-xL signaling, reducing B-cell activation and survival and suggesting a potential therapeutic strategy to reduce B-cell-mediated atherosclerotic inflammation [167]. These findings were confirmed in *Apoe*^*−/−*^ mice treated with PIP-CD47. Targeted BTK inhibition in macrophages and B cells lowered inflammation, stabilized plaques, and reduced atherosclerosis, improved the LDL/HDL profile, and caused no changes in weight or organ damage, indicating a safe therapy for atherosclerosis [167].

#### CSF-1R

The class III receptor tyrosine kinase colony-stimulating factor 1 receptor (CSF-1R) is identical to the cellular feline McDonough Sarcoma oncogene (c-fms) proto-oncogene [204, 205]. It is activated by the ligands CSF-1, also known as macrophage-CSF (M-CSF), and IL-34 [206], is expressed at low levels on hematopoietic stem cells [207], and at high levels on monocytes, macrophages [208, 209], osteoclasts, dendritic cells [210], as well as on various epithelial [211], and neurogenic cells [212, 213]. Concerning cellular processes, CSF-1R is involved in development, immunity, inflammation, tissue repair, and the tumor microenvironment [214, 215]. Particularly during inflammatory processes, CSF-1R regulates the survival [216], proliferation [217, 218], differentiation [219], and chemotaxis [220, 221] of macrophages, influencing their persistence within inflammatory lesions. Furthermore, bioinformatic analysis identified CSF-1R as a diagnostic marker of atherosclerosis [222] and found it at low levels in carotid atherosclerotic lesions [223]. Beyond vascular inflammation, elevated CSF-1R expression in adipose tissue macrophages inversely correlates with circulating HDL-C levels, linking CSF-1R-mediated macrophage activation to systemic metabolic inflammation and cardiovascular risk [224].

Investigations on clonal hematopoiesis (CH) focusing on IL-6 signaling in mouse and human ten-eleven translocation methylcytosine dioxygenase 2 (*Tet2*)-deficient macrophages show that IL-6 increases CSF-1R expression and promotes macrophage survival. In vivo, IL-6R blockade in *Tet2*-CH-*Ldlr*^*−/−*^ mice reduces plasma SAA, reverses monocytosis, attenuates the increase in aortic root lesion area, reduces plaque macrophage burden, enlarges the fibrous cap, and lowers CSF-1R expression in lesional macrophages and BMDMs. In vitro, anti-IL6R similarly lowers CSF-1R in BMDMs. CSF-1R expression is elevated at baseline and becomes strongly IL-6-inducible in *Tet2*-deficient cells. Mechanistically, *Tet2* loss increases nuclear signal transducer and activator of transcription 3 (STAT3) acetylation, thereby enhancing STAT3 binding to the CSF-1R promoter and increasing CSF-1R transcription. Pharmacologic CSF-1R inhibition with PLX3397 abolishes the apoptosis resistance of *Tet2*-deficient macrophages, normalizes PI3K/Akt signaling, reduces monocytosis, and reverses accelerated atherosclerosis, thereby lowering plaque macrophage content [168]. CSF-1R, as a key downstream factor of IL-6, was confirmed in vivo in *Tet2*-CH-*Ldlr*^*−/−*^ mice, and the CSF-1R inhibitor PLX3397 effectively neutralized the pro-atherogenic consequences of *Tet2*-deficient clones. Furthermore, human embryonic stem cell (ESC) and induced pluripotent stem cell (iPSC) models recapitulated the IL-6/CSF-1R/PI3K/Akt signaling axis, confirming that *Tet2* deficiency similarly enhances CSF-1R expression and macrophage survival in human cells [168].

#### EGFR

The epidermal growth factor receptor (EGFR) is a receptor tyrosine kinase (RTK) involved in various cellular processes in the vascular system, including atherosclerosis. The receptor can be activated by specific ligands such as EGF or heparin-bound EGF, or transactivated by other signals. EGFR expression is particularly abundant in macrophages, SMCs, ECs, and cardiomyocytes, which can also release EGFR ligands. It is already known that EGFR is involved in the transformation of macrophages into foam cells, in the proliferation of vascular SMCs, and in the amplification of inflammatory processes [225]. This includes the downstream activation of transcription factors such as NF-κB, which enhances the expression of proinflammatory genes in macrophages [226]. EGFR also mediates oxidative stress in macrophages via oxLDL [227], particularly when activated by the metalloproteinase meprin-α [228]. Inhibition of EGFR in a type 1 diabetes mouse model has been shown to prevent cardiac damage and myocardial structural remodeling, accompanied by reduced oxidative stress [169, 229]. Furthermore, results from a primate model suggest that EGF-related ligands may serve as markers for active inflammatory stages of atherosclerosis [169, 230].

The mechanisms of EGFR activation in atherosclerosis were analyzed in *Apoe*^*−/−*^ mouse models. After 8 weeks of HFD, these mice rapidly developed atherosclerotic lesions characterized by macrophage and SMC accumulation, foam cell formation, and elevated levels of inflammatory mediators, including IL-6, ICAM-1, TNF, and VCAM-1. At the same time, EGFR phosphorylation and activity increased, accompanied by elevated ROS levels and increased expression of MMP-2 and MMP-9. Treatment with EGFR inhibitors, such as AG1478 or compound 542, markedly reduced these atherosclerotic changes without altering serum LDL levels [169]. Furthermore, in oxLDL-treated primary macrophages, EGFR phosphorylation triggered NF-κB-dependent signaling. It elevated TNF, IL-6, ICAM-1, VCAM-1, MMP-2, and MMP-9 expression and activity, as well as ROS production, foam cell formation, and mitochondrial dysfunction. Pretreatment with the EGFR inhibitors AG1478 or compound 542 reversed these effects, as observed in the in vivo model [169]. OxLDL-induced EGFR activation in macrophages involved toll-like receptor 4 (TLR4). In WT macrophages, oxLDL induced cellular sarcoma (c-SRC) activation, followed by EGFR phosphorylation and subsequent AKT/ERK signaling. In contrast, in *Tlr4*^*−/−*^ macrophages, no EGFR phosphorylation or downstream inflammatory responses occurred. Inhibition of TLR4 with ethyl (6R9-6-[N-(2-chloro-4-fluorophenyl)sulfamoyl]cyclohex-1-ene-1-carboxylate (TAK242) or SRC with 4-amino-5-(4-chlorophenyl)-7-(t-butyl)pyrazolo[3,4-d]pyrimidine (PP2) also prevented EGFR activation. Thus, EGFR is activated by TLR4/c-SRC-dependent transactivation following stimulation with oxLDL [169]. Moreover, additional investigations on the EGFR mechanism identified golgi membrane protein 1 (GOLM1) as an inflammation-responsive factor that activates EGFR in macrophages. The ERK signaling cascade, activated as a result, promoted LDL uptake, foam cell formation, and the release of inflammatory cytokines. Neutralization or genetic deletion of GOLM1 reduced atherosclerosis in *Apoe*^*−/−*^ mice, confirming that EGFR activation via extracellular mediators, such as GOLM1, contributes to vascular inflammation and plaque progression [231].

These results demonstrate a detrimental effect of EGFR activity on atherosclerosis and the effectiveness of the EGFR inhibitors AG1478 and compound 542 in reducing atherosclerotic changes. This highlights the potential therapeutic benefit of EGFR inhibitors, particularly given their current use as first-line therapy in oncology and their potential to complement established treatments such as statins, angiotensin-converting enzyme (ACE) inhibitors, or niacin [169].

#### EPHA2

The receptor tyrosine Ephrin type-A receptor 2 (EphA2) predominantly interacts with glycosylphosphatidylinositol (GPI)-anchored ephrinA ligands and is associated with cell proliferation, tumor growth, and metastasis. Beyond its role in cancer, EphA2 is linked to MI [232, 233], inflammation, and the regulation of leukocyte functions [234]. In addition, EphA2 promotes fibronectin deposition, activation of mitogenic signaling pathways (ERK1/2, AKT1), and SMC proliferation [235]. Elevated EphA2 expression has been detected in macrophages within atherosclerotic plaques [236]. In *Apoe*^−/−^ mice, EphA2 knockdown reduced inflammation, decreased plaque formation, and inhibited NF-κB-dependent signaling [235]. The specific EphA2 inhibitor ALW-II-41-27 suppresses cell survival and proliferation, reduces proinflammatory responses, and affects cholesterol transport, indicating therapeutic potential in atherosclerosis [170, 237]. Based on these findings, the effects of statins, inhibitors of 3-hydroxy-3-methylglutaryl-CoA reductase (HMGCR), were investigated on macrophages, addressing the paradoxical question of why statins can promote inflammation despite their anti-inflammatory reputation, and focused particularly on the resulting increase in EphA2 expression [238]. HFD-fed *Apoe*^*−/−*^ mice and the EphA2 inhibitor ALW-II-41-27 were used as in vivo models, along with additional methods, to investigate plaque formation, inflammation, and plaque stability. The findings consistently demonstrated that EphA2 inhibition reduced atherosclerosis and pro-inflammatory signaling, thereby enhancing plaque stability [170, 239]. Since previous studies have shown that statins can paradoxically promote macrophage inflammation [240], the effects of statins on EphA2 were investigated. Treatment with atorvastatin, simvastatin, or fluvastatin increased EphA2 expression [170]. Furthermore, treatment of macrophages (RAW264.7, murine BMDMs, THP-1) with different statins resulted in the upregulation of inflammation-associated genes, including NLRP3, IL-1β, TNF, EphA2, and annexin A2 (AnxA2). It was further found that atorvastatin reduced the binding activity of the transcriptional repressor Krüppel-like factor 4 (KLF4) to the EphA2 promoter, thereby suppressing histone acetylase 11 (HDAC11) expression and subsequently upregulating EphA2 transcription. Inhibition of EphA2, either by siRNA or ALW-II-41-27, reversed the statin-induced pro-inflammatory gene expression. Comparable effects were observed in hepatocytes (alpha mouse liver 12 (AML12), HepG2), SMCs (MOVAS), and T cells (Jurkat), whereas ECs (HUVECs) showed no significant response to statin treatment [170]. In vivo, atorvastatin administration in HFD-fed *Apoe*^*−/−*^ mice resulted in marked upregulation of EphA2 expression in the aorta, while silencing EphA2 reduced atherosclerotic plaque formation and lipid accumulation, but increased collagen deposition, indicating the formation of more stable plaques. Moreover, a positive correlation between EphA2 expression levels and aortic lesion area (pro-atherogenic effect) was observed. Combined EphA2 inhibition and atorvastatin treatment enhanced the anti-atherogenic impact, and mechanistic analysis revealed that atorvastatin activated NF-κB phosphorylation in plaque macrophages, peritoneal macrophages, and hepatic tissue, whereas EphA2 inhibition attenuated this activation [170].

Investigations on PBMCs confirmed the statin-induced increase in pro-inflammatory cytokine release (IL-1β, TNF, IL-6), supporting the murine findings. Collectively, an HDAC11/KLF4-EphA2-NLRP3/NF-κB axis has been identified as a novel pro-inflammatory signaling pathway contributing to statin-induced macrophage activation, and targeting EphA2 has been suggested to provide a therapeutic advantage in atherosclerosis. However, clinical validation is lacking, and future investigations employing monocyte/macrophage-specific *EphA2* knockout models are warranted [170].

Building on these results, another study [239] investigated the systemic and metabolic effects of EphA2 inhibition with the ALW-II41-27 inhibitor, with particular emphasis on the gut-liver-vessel axis. The role of the gut microbiota was also investigated, which metabolizes drugs, precursors, and bioactive molecules [241]. Microbial metabolites such as trimethylamine N-oxide have been shown to promote atherosclerosis progression [242], whereas short-chain fatty acids (SCFAs) and secondary bile acids (SBAs) exert cardioprotective effects [243]. Treatment with ALW-II-41-27 reduced oxidative stress and the production of inflammatory cytokines such as TNF, IL-6, IL-17, and ICAM-1 in colonic tissue [244], and was associated with reduced NF-κB/NLRP3-driven inflammatory signaling in both intestinal macrophages and plaque-associated macrophages, which was accompanied by a marked attenuation of plaque burden in HFD-fed *Apoe*^*−/−*^ mice. Consistent with these anti-inflammatory effects, ALW-II-41-27 also improved plaque stability, as reflected by smaller necrotic cores and increased collagen and SMC content in atherosclerotic lesions. The treatment also increased the abundance of SBA-producing gut bacteria, such as Enterococcus, Akkermansia, Eggerthella, and Lactobacillus, and elevated plasma SBA levels. In contrast, plasma and hepatic cholesterol levels were significantly decreased, and colonic inflammation was mitigated [239]. Mechanistically, these lipid changes were accompanied by increased hepatic expression of regulators of cholesterol efflux and bile acid synthesis, which is consistent with enhanced hepatic cholesterol efflux and bile acid production under EphA2 blockade. Furthermore, fecal microbiota transplantation (FMT) from ALW-II-41-27-treated HFD-fed *Apoe*^*−/−*^ mice into untreated atherosclerotic mice also reduced plaque burden, supporting a microbiota-dependent mechanism. Quantitative metabolomic analysis of coronary artery disease (CAD) patient samples revealed altered bile acid metabolism. It elevated EphA2 expression, which correlated with lower plasma levels of deoxycholic acid (DCA) and hyodeoxycholic acid (HDCA), which are further converted to SBAs [239]. Taken together, these results suggest that EphA2 inhibition by ALW-II-41-27 not only modulates NF-κB/NLRP3-mediated intestinal and plaque inflammation and hepatic cholesterol and bile acid handling but also restores gut microbiome balance and attenuates atherosclerosis, underscoring its therapeutic potential in CVD [239].

#### FES

The cytoplasmic protein tyrosine kinase feline sarcoma oncogene (FES), encoded by the feline sarcoma proto-oncogene (fps), exhibits high structural similarity to the kinase feline erythroblastosis virus-associated receptor (FER), both belonging to the same subfamily. FES is closely involved in regulating cell motility, proliferation, differentiation, survival, and inflammatory processes, and plays a role in leukocyte extravasation [245]. Since leukocyte extravasation is strongly linked to the progression of atherosclerotic plaques [246], it was further investigated in combination with single-nucleotide polymorphisms (SNPs) [171]. The investigation focused on SNPs located on chromosome 15q26.1, with particular emphasis on rs17514846, previously identified in genome-wide association studies as the strongest CAD-associated variant [247, 248]. Within this genomic region, rs17514846 occurs in linkage disequilibrium with additional SNPs (r^2^ ≥ 0.8) and lies in proximity to the furin gene (upstream) and the FES proto-oncogene [249]. It is well established that furin promotes atherosclerosis in murine models [250] and that its expression is influenced by the rs17514846 [249], thereby connecting the furin locus, the rs17514846 SNP, and atherosclerotic progression. The functional relevance of CAD-associated variants at 15q26.1 was assessed by examining their impact on FES expression. It aimed to clarify how FES function influences both the formation and cellular makeup of atherosclerotic plaques. It was revealed that FES acts as a protective factor against atherosclerosis and that disease-associated genetic variants at the 15q26.1 locus enhance cardiovascular risk by diminishing FES expression [171]. CRISPR-edited THP-1 monocytes demonstrated that the rs17514846-A and rs1894401-G risk alleles directly lower FES levels, while expression quantitative trait locus (eQTL) data support similar effects in vascular SMCs. Additional mechanistic insights were obtained from monocytes, where inhibition of the transcription factor positive regulatory domain I-binding factor 1 (PRDM1), which binds to rs1894401 in intron 3 of FES, increased histone H3 acetylation at the FES locus and elevated FES mRNA and protein expression [171]. Functional characterization using *Fes*^−/−^ (*Apoe*^*−/−*^) mice, alongside primary human monocytes with FES siRNA knockdown, confirmed the presence of larger, more macrophage-rich atherosclerotic lesions and increased monocyte migration (in primary cells) and proliferation (in THP-1 cells). This suggests that FES limits immune cell activation, thereby exerting a protective effect against excessive inflammation and atherosclerosis [171]. Consistent with the murine data, human coronary atherosclerotic plaques from rs17514846‑AA risk‑allele carriers show reduced FES expression in plaque macrophages and increased macrophage content compared with CC carriers, indicating that genetic variation at 15q26.1 translates into altered plaque composition in vivo. To delineate downstream pathways, phosphoproteomic profiling of FES-silenced monocytes identified 29 proteins with altered phosphorylation at tyrosine, serine, and threonine residues, with enrichment in pathways related to mRNA processing, the rho-related BTB-domain containing protein (RhoBTB)/RhBTB2 guanosin-triphosphatase (GTPase) cycle, and RNA polymerase II transcription termination, and mRNA processing [171]. Consistent with the murine results, human atherosclerotic plaques from carriers of risk genotypes exhibited increased macrophage content, indicating that genetic variation translates into altered plaque composition in vivo [171]. Furthermore, FES has been shown to inhibit SMC migration and plaque accumulation, as evidenced by increased SMC abundance in plaques of *Fes*^*−/−*^ mice and by enhanced migration of FES-silenced vascular SMCs in vitro [171].

#### FGFR1

The fibroblast growth factor receptor 1 (FGFR1) is a receptor tyrosine kinase that plays a crucial role in cell proliferation, differentiation, and tumorigenesis. Beyond its oncogenic relevance, FGFR1 contributes to inflammatory disease mechanisms, including cardiovascular inflammation [251–257]. Pharmacological inhibition or genetic deletion of FGFR1 has been shown to reduce systemic inflammation in experimental models [172, 258, 259].

With respect to atherosclerosis, FGFR1 and its ligands fibroblast growth factor 1 (FGF1) and FGF2 are expressed in macrophages and SMCs [260, 261], during both early and advanced stages of lesion development [262]. In HFD-fed *Apoe*^*−/−*^ mice, treatment with the non-selective FGFR inhibitor SU5402 reduced neointimal growth, suggesting a functional link between FGFR1 activity and atherosclerotic progression [172, 263]. To further elucidate the role of FGFR1 in atherosclerosis, a myeloid-specific FGFR1 deletion driven by lysozyme-C (LysM) expression in *Apoe*^*−/−*^ mice was investigated in comparison to control *Apoe*^*−/−*^ mice treated with the FGFR1 inhibitor AZD4547. The atherosclerotic lesion formation was markedly reduced in both models [172]. Furthermore, in murine macrophages, activation of FGFR1 by oxLDL stimulation induced the release of inflammatory cytokines and chemokines, activated phospholipase C gamma (PLCγ) and NF-κB, and enhanced oxLDL uptake [172]. Beyond NF-κB, further signaling proteins, including Rho GTPases, MAP kinases, and AKT, were activated by FGFR1. Collectively, these findings highlight a previously unrecognized FGFR1-PLCγ-NF-κB-dependent signaling pathway in the progression of atherosclerosis [172].

#### JAK1, JAK2, and JAK3

The non-receptor-associated protein tyrosine kinase Janus kinase 2 (JAK2), together with the additional cytoplasmic signaling proteins JAK1, JAK3, and tyrosine kinase 2 (TYK2), belongs to the JAK family. JAKs are activated by cytokine-mediated trans-phosphorylation, after which they phosphorylate cytokine receptors and STAT proteins [264, 265]. Subsequently, STAT proteins are recruited and activated by JAKs [266]. In addition to JAK2, JAK1 has also been implicated in atherosclerosis, where it modulates inflammatory responses and macrophage activation through STAT1 and STAT3-dependent signaling [267–270]. Likewise, JAK3 has been shown to mediate Oncostatin M-induced STAT3 activation and osteogenic differentiation of vascular SMCs, thereby linking this pathway to atherosclerotic calcification [271, 272]. Similarly, JAK2-dependent STAT-signaling pathways are crucial for macrophage function and vascular inflammation [273–275]. These pathways also regulate lipid-handling receptors and transporters in macrophages, including the scavenger receptor CD36, as well as the cholesterol transporters ABCA1 and ATP-binding cassette subfamily G member 1 (ABCG1) [276–278]. Inhibition of JAK2 reduces vascular inflammation and cell proliferation [279, 280], whereas its upregulation may drive atherosclerosis [281]. In addition to the classical variants of the JAK kinases [173], standard driver mutations, such as JAK2V617F (JAK2VF) in the pseudokinase domain of JAK2, can also have pathologic effects. JAK2VF was investigated in connection with myeloproliferative neoplasms (MPN) [282]. This mutation increases hematopoietic cytokine signaling and drives CH [283], which in turn is a significant genetic risk factor for CVD. JAK2VF-CH is so far most strongly associated with a 12.1-fold increased risk of CVD. In contrast, other more common mutations, such as those in DNA (cytosine-5)-methyltransferase 3 A (DNMT3A), TET2, or additional sex combs-like 1 (ASXL1), are associated with a 1.7- to 2.0-fold increased risk [174]. A hyperlipidemic low-allele burden (LAB) model of JAK2VF with atherosclerosis was employed to investigate the mechanisms underlying accelerated atherosclerosis. The LAB model was generated by transplanting 1.5% JAK2VF bone marrow together with 98.5% green fluorescent protein (GFP)^+^ WT bone marrow into *Ldlr*^*−/−*^ mice after lethal irradiation, followed by a 16-week WD to induce atherosclerotic lesions [173]. The focus of this study was particularly on IL-1β-mediated crosstalk between mutated and WT myeloid cells, resulting in reduced levels of MerTK and triggering receptor expressed on myeloid cells 2 (TREM2). In advanced atherosclerotic plaques, the efferocytosis receptor MerTK in macrophages acts stabilizing by reducing necrotic cores and strengthening the fibrous cap [284]. TREM2, on the other hand, functions as a phagocytic receptor that supports the survival of microglia and macrophages and promotes the activation of genes involved in efferocytosis, lysosomal degradation, and cholesterol transport [285, 286]. It was demonstrated that improved plaque stability was achieved by stabilizing MerTK and TREM2, and that as few as 1.5% mutated JAK2VF cells were sufficient to cause pronounced atherosclerosis [173]. The LAB model developed more severe atherosclerosis, with lesion area enlarged by about 50%, necrotic cores nearly doubled in size, fibrous caps reduced, and collagen content unchanged. Furthermore, an increase in macrophages in the lesions was measured, with the proportion of mutant to WT macrophages increasing. The moderate expansion of mutated macrophages accounted for about 7% of total macrophages. With respect to inflammatory processes, neutrophil extracellular traps (NETs) in the lesions increased, as did the inflammatory cytokines IL-1β in mutated and WT macrophages, and IL-6. Thus, even a small proportion of JAK2VF macrophages in plaques contributes to a significant increase in atherosclerosis and plaque instability, with clear evidence of inflammatory crosstalk from mutated to WT cells, mainly mediated by IL-1β [173]. The crosstalk was further examined in IL-1 receptor 1-deficient (*Il1r1*^−/−^) mice with non-JAK2VF bone marrow cells. The increase in plaque size and necrotic area, as well as the reduction in fibrous cap, were entirely or partially reversed, along with the decrease in the phagocytic receptors MerTK and TREM2 [173]. Based on the findings regarding the mutated form of the kinase JAK2, shared mechanisms of inflammasome activation in TET2-, JAK2VF-, and probably ASXL1-CH may explain the inflammatory crosstalk, and targeted therapies against inflammasomes and their effectors could offer new treatment approaches for patients with clonal hematopoiesis [173]. In addition to genetically activated JAK2 variants such as JAK2VF, which promote CH and atherosclerosis, pharmacological research also highlights the central role of JAK2. The selective JAK2 inhibitor fedratinib reduced the expansion of hematopoietic stem and progenitor cells, myelopoiesis, and plaque formation without affecting cholesterol levels. Therefore, not only mutated but also hypercholesterolemia-driven JAK2 signaling contributes to atherogenesis [175].

#### Lyn

The non-receptor tyrosine kinase of the Src family, Lck/Yes-related novel tyrosine kinase (Lyn), is predominantly expressed in B lymphocytes and various myeloid lineages, and is also detectable in neural and epithelial tissues [287–289]. It modulates the signal transduction of immune receptors such as B-cell receptor (BCR), fragment crystallizable (Fc) receptors [290], integrins [291], and TLRs [292], and mediates both pro-inflammatory and immunosuppressive signals [289]. Two isoforms of Lyn exist: LynA, with an activating focus, and LynB, which acts mainly inhibitory, generated by alternative splicing. In addition to inflammation, the kinase Lyn has been linked to autoimmune diseases, such as systemic lupus erythematosus, and to cancer [289, 293]. Lyn has been identified as a central prognostic factor in atherosclerosis and as an indicator of the risk of ischemic insults [29]. In vitro investigations of BMDMs and in vivo investigations of *Apoe*^*−/−*^ mice demonstrated an interaction between Lyn-AKT and sorting nexin 10 (SNX10). *Apoe*^*−/−*^ mice with a myeloid-specific *Snx10* knockout showed reduced Lyn activation and, as a result, decreased AKT phosphorylation. In BMDMs, oxLDL uptake triggered the CD36-Lyn-AKT signaling cascade, with SNX10 required for Lyn and AKT recruitment and interaction. In the absence of SNX10, oxLDL uptake was significantly reduced, accompanied by increased activation of the transcription factor EB (TFEB), resulting in enhanced lysosomal biogenesis, lipolysis, and fatty acid oxidation. Typically, AKT, activated through the CD36-Lyn-AKT signaling cascade, inhibits the translocation of TFEB into the nucleus. The macrophages were metabolically reprogrammed and shifted toward an anti-inflammatory phenotype. In *Apoe*^*−/−*^ mice, myeloid-specific *Snx10* knockout resulted in a marked reduction in atherosclerotic lesion formation. Therefore, SNX10 is a potential therapeutic target for atherosclerosis [176].

#### MerTK

The macrophage surface protein Mer proto-oncogene tyrosine kinase (MerTK) mediates efferocytosis and anti-inflammatory signaling. MerTK is activated by the ligands growth arrest-specific 6 (Gas6) and Protein S, which bind to phosphatidylserine on apoptotic cells [294, 295]. Through this interaction, MerTK promotes the uptake of apoptotic cells and triggers anti-inflammatory responses [296, 297]. Under inflammatory conditions, MerTK expression contributes to the resolution of inflammation and is associated with anti-inflammatory macrophage phenotypes [298–300]. Conversely, disrupted blood flow reduces MerTK expression and impairs efferocytosis [301, 302]. Additionally, system-wide transcriptomic data indicate high MerTK expression in cardiovascular tissues, suggesting a protective role in this context [303]. Moreover, MerTK can undergo proteolytic cleavage, releasing soluble Mer that can interfere with ligand binding and thereby modulate MerTK signaling [304, 305].

In atherosclerosis, MerTK activity plays a crucial role in determining plaque stability, serving as a key receptor for efferocytosis, and is impaired under hypoxic conditions [306]. However, it can be enhanced by pharmacological or nanotechnological interventions that upregulate MerTK expression [307–309]. In HFD-fed *Apoe*^*−/−*^ mice treated with streptozotocin to induce diabetes, it was revealed that diabetes accelerates atherosclerosis progression and that elevated glucose levels impair efferocytosis. Consistently, BMDMs and RAW264.7 cells exhibited reduced MerTK expression under high-glucose conditions compared with normal-glucose conditions. Thus, reduced MerTK levels directly translate into diminished efferocytosis capacity [177]. Subsequently, hybrid membrane nanovesicles (HMNVs) were generated by fusing membranes from RAW264.7 cells overexpressing MerTK with membranes from human embryonic kidney 293T cells (HEK293T cells) overexpressing the transferrin receptor (TfR). They were evaluated as a strategy to restore MerTK expression. For optimization, vesicles were further modified with 1,2-dioleoyl-sn-glycero-3-phosphoethanolamin (DOPE). This lipid promotes membrane fusion, and when combined with superparamagnetic iron oxide nanoparticles (SMN), enables targeted navigation and in vivo tracking via magnetic field application. These HMNVs effectively improved macrophage efferocytosis [177]. The vesicles were then administered intravenously to streptozotocin-treated *Apoe*^*−/−*^ mice with established diabetes and atherosclerosis. HMNVs accumulated predominantly in the liver and spleen and were also detectable in the aorta. Treated mice exhibited a reduced plaque burden and smaller necrotic cores. At the same time, the collagen content remained unchanged, indicating that the plaque stability was preserved. There were no alterations in glucose or lipid levels, and arterial stiffness remained unaffected. These findings demonstrate that HMNVs attenuate atherosclerosis in diabetic *Apoe*^*−/−*^ mice, independently of metabolic parameters [177]. In conclusion, elevated glucose directly decreases MerTK expression at both the RNA and protein levels, thereby exacerbating atherosclerosis in diabetes. Notably, the impaired efferocytosis could be reversed and the plaque burden reduced by administering MerTK-loaded hybrid membrane nanovesicles, with no observable adverse side effects [177].

#### FAK, and PYK2

Focal adhesion kinase (FAK), encoded by the protein tyrosine kinase 2 (PTK2) gene, is a non-receptor tyrosine kinase localized at focal adhesions [310]. It is involved in cell adhesions, migration, proliferation, and survival, and functions as a mediator of integrin and growth factor signaling. In the intestinal epithelium, FAK is essential for epithelial restitution and sheet migration, coupling to the ERK/MAPK, PI3K-AKT, and CT10 regulator of kinase-associated substrate (p130Cas-Crk) signaling pathways [311]. Moreover, FAK contributes to immune cell functions, including macrophage chemotaxis [312]. FAK has previously been associated with intestinal homeostasis and chronic inflammatory bowel diseases, as well as with the regulation of inflammatory signaling pathways in vascular ECs [178, 311]. However, FAK has been shown to exert secondary effects on macrophage recruitment by modulating endothelial inflammatory signaling. Investigations on human aortic endothelial cells (HAoECs) have demonstrated that FAK and the related kinase protein proline-rich tyrosine kinase 2 (PYK2) are crucial for the TNF- and IL-1β-induced expression of pro-inflammatory adhesion molecules, including VCAM-1, ICAM-1, and E-selectin. Pharmacological inhibition of FAK/PYK2 with PF-271 and VS-6063 or targeted knockdown of FAK or PYK2 reduced their activity or expression, thereby decreasing monocyte adhesion and transmigration [178]. Furthermore, activation of ERK and JNK MAPKs, mediated by FAK/PYK2 activity, was observed; however, early NF-κB activation, which occurred within 60 min, remained largely unaffected. In addition, it has been demonstrated that FAK/PYK2 regulates the transcription of other pro-inflammatory genes, including monocyte chemoattractant protein-1 (MCP-1, also known as CCL2), interferon-γ-induced protein-10 (IP-10), and C-X-C motif chemokine ligand 11 (CXCL11). The observed effects were subsequently confirmed in the *Apoe*^*−/−*^ mouse model of atherosclerosis, using induced carotid ligation and an HFD. Pharmacological inhibition of FAK/PYK2 reduced vascular VCAM-1 expression in the mice and prevented the recruitment of macrophages to the vessel wall. Therefore, FAK/PYK2 inhibitors are highlighted as potential therapeutics for the treatment of atherosclerosis [178].

#### SYK

The non-receptor spleen tyrosine kinase (SYK) is predominantly expressed in hematopoietic cells. Still, it is also present in non-hematopoietic tissues such as normal mammary glands and mammary epithelial cell lines [313, 314]. SYK mediates signal transduction downstream of B-cell receptors and Fc receptors, as well as integrin signaling in neutrophils, macrophages, and platelets [315, 316]. Through these pathways, SYK is implicated in allergic and autoimmune diseases, including rheumatoid arthritis and immune thrombocytopenia [179, 180, 315, 316]. Additionally, SYK regulates inflammatory signaling in macrophages and dendritic cells in atherosclerosis. In line with this role, SYK also regulates inflammatory signaling pathways in macrophages and dendritic cells during atherosclerosis [179, 180]. Cholesterol crystals, C-reactive protein (CRP), and platelet-derived soluble TREM-like transcript 1 (TLT1) activate SYK-dependent PI3K, ERK, and NF-κB pathways, promoting cytokine release and plaque inflammation [317–320]. SYK also signals downstream of the collagen receptor glycoprotein VI (GpVI) and C-type lectins, enabling the recognition of fungi, microbial pathogens, and tissue damage and activating the caspase recruitment domain protein (CARD) – B-cell lymphoma/leukemia 10 protein (BCL10) – mucosa-associated lymphoid tissue lymphoma translocation protein 1 (MALT1) (CARD-BCL10-MALT1) pathway [321, 322]. Moreover, SYK is required for activation of the NLRP3 inflammasome after fungal infection [323]. The SYK inhibitor fostamatinib was used to investigate the effects of SYK inhibition in *Ldlr*^*−/−*^ mice on a HFD. Several investigations have demonstrated that the administration of fostamatinib reduces the recruitment and migration of immune cells to sites of inflammation, decreases the number of macrophages in plaques, attenuates inflammatory cytokine production, and impairs macrophage differentiation and survival. Histological quantification of lesion size in the aortic root and arch, together with blood cell analyses focusing on monocytes, neutrophils, and lymphocytes of *Ldlr*^*−/−*^ mice, revealed a fostamatinib dose-dependent reduction in plaque size. Further plaque analysis showed fewer macrophages, increased collagen and SMCs, decreased IL-6 expression, and a trend toward lower IL-12 and TNF levels. Altered cytokine expression under SYK inhibition was confirmed in TNF- or LPS-stimulated ECs treated with the active SYK inhibitor metabolite R406 [179]. To assess whether fostamatinib also modulates acute systemic inflammation, *Ldlr*^*−/−*^ mice were injected intraperitoneally with TNF (10 µg/kg) or LPS (1 mg/kg). Under these conditions, fostamatinib reduced TNF-induced IFN-γ and LPS-induced IL-12 and IFN-γ levels, and decreased leukocyte recruitment, accompanied by a shift from granulocyte marker 1 (Gr-1)^high^ to Gr-1^low^ monocytes, indicating a systemic anti-inflammatory effect of SYK inhibition [179]. The reduced number of macrophages could be attributed to impaired cell recruitment induced by fostamatinib. Intravital microscopy in HFD-fed mice treated with fostamatinib demonstrated reduced ICAM-1 and VCAM-1 expression along the plaque endothelium [179]. Furthermore, SYK blockade impaired M-CSF–dependent survival signaling and reduced both the proportion of macrophages and their F4/80 expression, indicating diminished macrophage differentiation [179]. In conclusion, SYK inhibition impairs macrophage differentiation and survival, reduces the numbers of macrophages and leukocytes, exerts anti-inflammatory effects, and prevents plaque progression by the transmigration of inflammatory cells. Therefore, there is evidence for the potential application of fostamatinib in atherosclerosis to reduce and stabilize plaques and dampen the inflammatory response [179]. However, it is important to note that there are indications that these pro-inflammatory functions of SYK are at least to some extent context-dependent, as SYK activation has also been associated with increased phagocytosis and efferocytosis capacity, which would reflect a more M2-like macrophage phenotype [324].

Further investigations examined pathological activation of SYK following MI in *Apoe*^*−/−*^ mice and in chimeric mice generated by adoptive bone marrow transfer. It has been demonstrated that increased SYK signaling in CVD arises from MI-induced trained immunity, driven by lysine methyltransferase 5 A (Kmt5a) / CCHC-Type Zinc Finger Nucleic Acid Binding Protein (CNBP) and sympathetic signaling [180]. Furthermore, it was shown that MI promotes myeloid cell infiltration into plaques and accelerates atherosclerosis, as demonstrated by bone marrow transplantation from MI versus sham donors into naive *Apoe*^*−/−*^ recipients. Elevated SYK expression was detected in *Apoe*^*−/−*^ mice with MI or ischemia-reperfusion, particularly in plaque-associated macrophages, establishing SYK as a marker of pro-inflammatory activation [180]. These findings were further validated in BMDMs and hematopoietic stem cells (HSCs) from MI mice, revealing associations with trained immunity and epigenetic regulation. SYK inhibition with histone methyltransferase inhibitors (UNC0379) increased *Kmt5a* expression and enriched H4K20me on the SYK gene. MI-BMDMs stimulated with oxLDL exhibited increased SYK/Kmt5a/H4K20me, indicating MI-induced epigenetic imprinting of monocytes [180]. Analysis of MI-associated trained immunity revealed that CNBP functions as a transcriptional partner of *Kmt5a*. Following MI, CNBP expression was elevated in monocytes and macrophages, and together with Kmt5a, it bound to the SYK gene, enhancing SYK transcription. This established the CNBP/Kmt5a complex as a key epigenetic regulator of MI-induced immune training. Consistent with this role, silencing either *Kmt5a* or *Cnbp* in *Apoe*^*−/−*^ MI mice reduced plaque burden, lowered SYK expression, decreased pro-inflammatory gene levels, and diminished H4K20me, demonstrating the functional importance of this regulatory axis in post-MI atherogenesis [180]. Furthermore, in patients with ST-elevation myocardial infarction (STEMI), classical monocytes exhibited significantly increased SYK and Kmt5a expression, with SYK correlating with plaque burden (Gensini score) and elevated serum levels of IL-1β and TNF. These findings highlight SYK as a potential biomarker for atherosclerosis progression in STEMI patients [180].

#### TNK1

The non-receptor-associated tyrosine kinase tyrosine kinase nonreceptor 1 (TNK1) belongs to the activated Cdc42-associated kinase (ACK) family and regulates TYK2 activation within the JAK/STAT signaling pathway. TNK1 expression has been identified in B-lymphomas, hepatocytes, and various tumor cell types [325, 326] and has been linked to several infectious processes. For instance, during hepatitis C virus (HCV) infection, TNK1 is recruited and phosphorylated, thereby activating the TYK2/STAT1 signaling pathway. Activated STAT1 induces the expression of more than 300 IFN-stimulated genes and contributes to the control of HCV infection [181, 325]. However, TNK1 has also been associated with atherosclerotic inflammation. It has been reported that TNK1 expression is significantly increased in the aorta of HFD-fed *Apoe*^*−/−*^ mice [181]. At the same time, a similar finding was present in human carotid endarterectomy (CEA) samples. Notably, ruptured plaques in CEA samples exhibited significantly higher TNK1 expression than stable plaques [181]. At the cellular level, THP-1 monocytes/macrophages exhibited substantially higher TNK1 expression than HUVECs, human brain microvascular endothelial cells (HBMECs), and human aortic vascular SMCs (HA-VSMCs), all of which are relevant cell types in atherosclerosis. Stimulation of THP-1-derived macrophages with oxLDL resulted in pronounced upregulation of TNK1 and increased secretion of pro-inflammatory cytokines, including IL-6, IL-12, and TNF. By specifically inhibiting TNK1 expression using shRNA (shTNK1), both the inflammatory response and the lipid uptake, as well as the intracellular cholesterol content in THP-1 cells, were reduced. Mechanistically, TNK1 has been shown to promote pro-inflammatory signaling by phosphorylating TYK2 and STAT1 [181].

### TKL Group

**Fig. 9:**
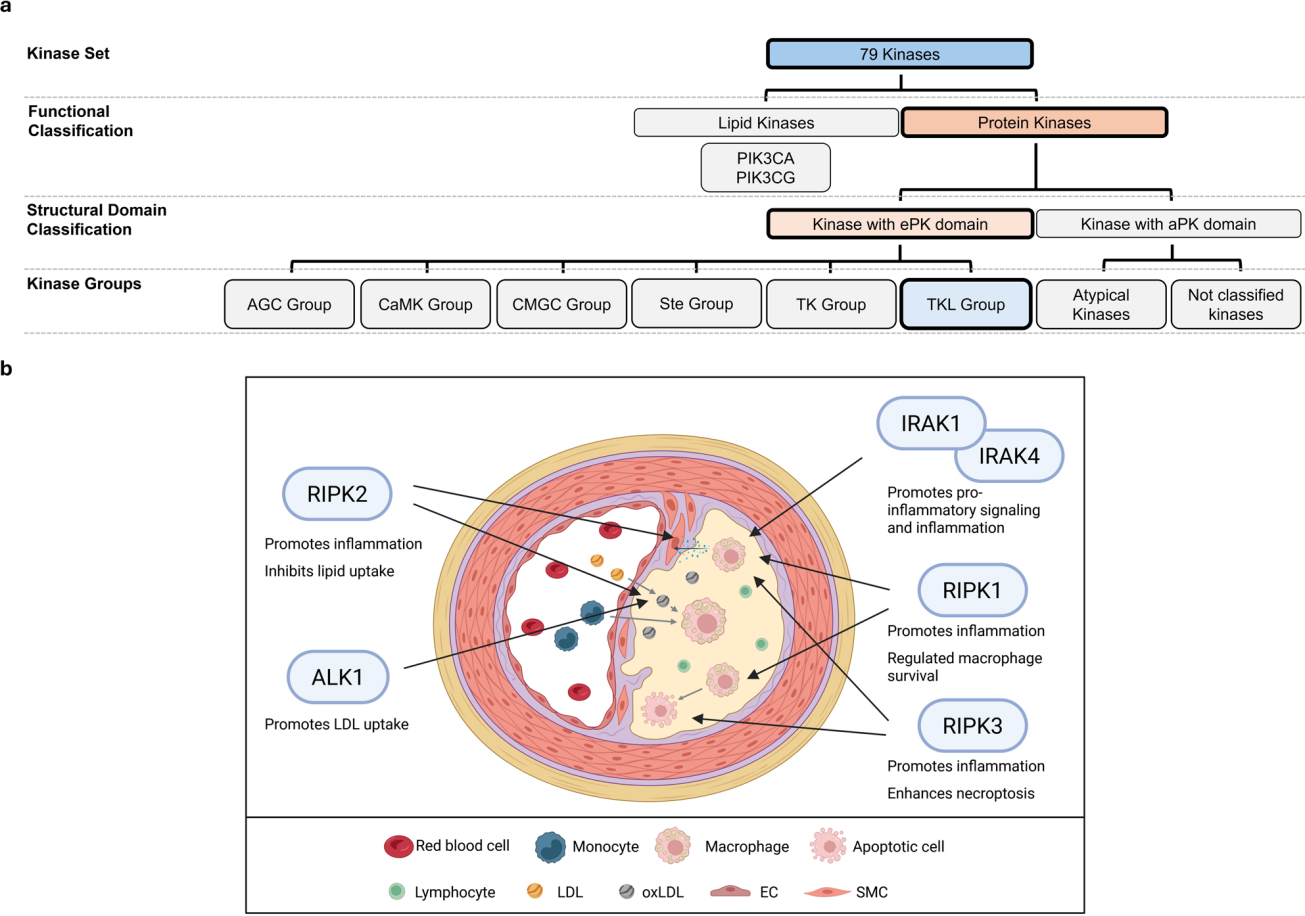
Overview of the role of the TKL Group of kinases in atherosclerosis. **a** Classification of the 76 kinases into major structural and functional groups, highlighting the TKL Group. **b** Illustration of the cellular microenvironment of an atherosclerotic lesion and visualizing the involvement of TKL Group kinases. EC: endothelial cell; LDL: low-density lipoprotein; oxLDL: oxidized LDL; SMC: smooth muscle cell

The tyrosine kinase-like (TKL) group is structurally similar to the TK group and encompasses tyrosine kinase members and serine/threonine kinases. That is why these kinases play a role in tyrosine- and serine/threonine-signaling pathways, including the MAPK cascade, immune and inflammatory signaling, apoptosis and survival signals, and cytoskeletal organization [99]. TKL kinases such as ALK1, IRAK1, IRAK4, RIPK1, RIPK2, and RIPK3 were associated and further investigated in their functions in atherosclerosis [327–333], while other kinases of this group, like LIMK1, TGFBR1, TGFBR2, MLKL, and TKL have been only peripherally associated with atherosclerosis and remain insufficiently characterized concerning their potential mechanistic involvement in the disease’s pathogenesis [334–337] (Fig. 9).

#### ALK1

The endothelial receptor activin-like kinase receptor 1 (ALK1, also known as ACVRL1), in conjunction with the Srb1, is responsible for LDL transcytosis across the endothelium. Several studies have shown that LDL entry via these receptors occurs independently of the classical LDL receptor [338–340]. After elevated ALK1 mRNA levels were detected in human atherosclerotic arteries and in *Ldlr*^*−/−*^ mice, ALK1 was specifically knocked out in arterial ECs. To achieve this, *Alk1*^*f/f*^ mice were crossed with bone marrow tyrosine kinase on chromosome X cre-recombinase estrogen receptor 2 (*Bmx-Cre-ERT2*) mice (tamoxifen-inducible Cre recombinase under the *Bmx* promoter) on a membrane-targeted tomato membrane-targeted GFP (mTmG) reporter background (*Alk1*^*iΔaEC*^), enabling selective and inducible deletion of *Alk1* in arterial endothelium without affecting venous or capillary ECs. Subsequently, *Alk1*^*iΔaEC*^ mice in which LDLr was functionally deleted via AAV-PCSK9-injection (constituting a PCSK9-AAV hypercholesterolemia model) exhibited no changes in lipid profiles or body weight, but showed a marked reduction in neutral lipid content within the aorta and decreased macrophage infiltration [327]. Similarly, dKO mice *Alk1*^*iΔaEC*^ *Ldlr*^−*/*−^ displayed unchanged blood pressure and plasma lipid levels, but showed significantly reduced LDL uptake and ApoB100/48 accumulation in the aorta, as well as decreased plaque size, macrophage infiltration, lipid deposition, and collagen content. These findings suggest that ALK1 plays a crucial role in endothelial LDL transcytosis and atherogenesis, independent of LDLr. At the signaling level, ALK1 functions as a receptor for bone morphogenetic protein-9/10 (BMP9/10), activating SMAD1/5 in ECs. Based on these results, a selective monoclonal anti-ALK1 antibody (mAb2) was developed, which inhibits LDL uptake and transcytosis without interfering with the BMP9/10–ALK1–SMAD1/5 signaling pathway. The efficacy of mAb2 was evaluated both in vitro (HUVECs, human coronary artery endothelial cells (HCAECs), and mouse lung endothelial cells (MLECs)) and in vivo (*Ldlr*^−*/*−^ mice). It was demonstrated that mAb2 binds to ALK1 with high affinity and effectively inhibits LDL transcytosis, highlighting its therapeutic potential [327]. Consistent with the findings in *Alk1*^*iΔaEC*^ *Ldlr*^−*/*−^ mice, in vivo treatment of *Ldlr*^−*/*−^ mice with mAb2 markedly reduced atherosclerotic lesion formation, ApoB accumulation, macrophage infiltration, and collagen deposition, suggesting that neutralization of ALK1 prevents or attenuates atherosclerotic progression. Furthermore, accelerated plaque regression under lipid-lowering conditions was observed in *Ldlr*^−*/*−^ mice fed a 12-week WD and subsequently switched to a standard chow diet for 4 weeks with mAb2 treatment, compared with untreated controls [327]. These findings underscore the central role of the endothelial transcytosis receptor ALK1 in atherosclerosis and suggest that therapeutic inhibition of ALK1 with mAb2 could be a promising strategy to complement existing lipid-lowering therapies, such as statins or PCSK9 inhibitors, although mAb2 is currently a preclinical agent with no clinical trials reported yet.

#### IRAK1 and IRAK4

The IL-1 receptor-associated kinase (IRAK) family includes the four kinases IRAK1, IRAK2, IRAK-M, and IRAK4. These kinases mediate intracellular signaling via the adaptor protein myeloid differentiation primary response 88 (MyD88), acting downstream of TLRs and IL-1Rs. Through this pathway, IRAKs contribute to the activation of signaling cascades, including NF-κB, MAPK, PI3K-AKT-mechanistic target of rapamycin (mTOR), JAK/STAT, interferon regulatory factor (IRFs), and activator protein 1 (AP-1), thereby controlling the expression of cytokines and inflammatory responses. While IRAK1, IRAK2, and IRAK4 are ubiquitously expressed across tissues, IRAK-M is expressed only in monocytes and macrophages [341]. The kinase IRAK4 activates IRAK1, which functions as a signal amplifier and integrator [342]. Its activity has been linked to NF-κB-dependent inflammatory signaling and cholesterol metabolism in macrophages, highlighting its potential relevance for atherosclerosis [343, 344]. Similarly, the critical role of IRAK4 in atherosclerosis has been demonstrated in various genetic model studies. For example, genetic inactivation of IRAK4 kinase activity in *Apoe*^*−/−*^ mice resulted in a pronounced inhibition of plaque formation following carotid ligation [330]. Moreover, both macrophage and SMC accumulation in the lesions were decreased after ligation. These findings identify IRAK4 as a therapeutic target of vascular inflammation and plaque development. Consistently, a downregulation of key inflammatory genes, including MCP-1, IL-6, lipocalin 2 (Lcn2), and subunits of nicotinamide adenine dinucleotide phosphate (NADPH) oxidase, was detected and was associated with an inhibition of macrophage accumulation [330]. Another study confirmed that IRAK4 plays a pivotal role in atherosclerosis formation and modified LDL-mediated signaling [329]. Further investigations revealed that the interaction of IRAK1 with TRAF6 is negatively regulated by the major vault protein (MVP). Loss of *Mvp* in HFD-fed mice resulted in increased plasma lipid levels and aggravated atherosclerosis in *Apoe*^*−/−*^ mice. This was associated with increased TRAF6 recruitment to IRAK1 and enhanced NF-κB-mediated inflammatory signaling. Consequently, these animals developed more severe atherosclerosis and obesity, whereas MVP overexpression suppressed inflammatory signaling. Together, these findings suggest a contribution of IRAK1-dependent signaling to atherogenic inflammation within a pro-atherogenic TLR-TRAF6-NF-κB-dependent signaling axis [328].

#### RIPK1 and RIPK3

Receptor-interacting protein kinase 1 (RIPK1) and RIPK3 are serine/threonine protein kinases that regulate innate immune and cellular stress signaling pathways [345, 346]. Following TNF receptor 1 (TNFR1) activation, RIPK1 is recruited to signaling complex I and acts as a molecular switch determining whether a cell activates survival pathways or undergoes programmed cell death [347, 348]. When ubiquitinated, RIPK1 promotes NF-κB-dependent survival signaling [347]. In contrast, the loss of ubiquitination shifts the signaling pathway toward apoptosis or necroptosis [349]. Under specific conditions, such as impaired ubiquitination or caspase inhibition, RIPK1 activates RIPK3, triggering the necroptotic cascade [349, 350]. RIPK3 promotes MLKL phosphorylation, leading to membrane rupture, the release of damage-associated molecular patterns (DAMPs), and a robust inflammatory response [350, 351]. RIPK1/RIPK3-mediated necroptosis has been identified in human atherosclerotic plaques and is associated with the formation of necrotic cores, particularly in unstable lesions. In macrophages, RIPK1 and RIPK3 link dysregulated lipid handling with inflammatory activation, thereby promoting atherosclerotic progression [337, 352–354]. It is well established that inhibiting RIPK3 phosphorylation or genetically deleting *Ripk3* reduces macrophage necroptosis and necrotic core formation in atherosclerotic plaques, thereby enhancing plaque stability. This relationship was further examined in human and murine models, which confirmed that necroptosis is the dominant cell-death mechanism in unstable plaques and that RIPK3 expression correlates with necrotic core development [332]. Furthermore, a negative feedback loop was identified in which RIPK3 induces miR-223-3p transcription via C/enhancer binding protein β (EBPβ), which, in turn, inhibits RIPK3 and thereby limits necroptosis of foam cells. Deletion of *Mir223* in hematopoietic cells of *Apoe*^*−/−*^ mice increased atherosclerosis, an effect reversed by *Ripk3* deficiency or pharmacological inhibition of RIPK1/RIPK3 (Necrostatin-1 or GSK-872). These findings suggest therapeutic potential for microRNA-based modulation of necroptosis in atherosclerosis [332]. The function of RIPK1 in atherosclerosis was further investigated in human atherosclerotic tissue and in complementary mouse and cellular models. RIPK1 expression was elevated in human coronary and carotid plaques and correlated with NF-κB-dependent inflammatory markers, including IKK and TNF [331]. Furthermore, it has been demonstrated that RIPK1 activity is essential for triggering NF-κB activation and pro-inflammatory cytokine expression in macrophages. This effect was confirmed in vivo in NF-κB reporter mice and was also observed in HUVECs. The HUVEC experiment also indicated a pro-inflammatory role for RIPK1 in inflammatory adhesion, as RIPK1 knockdown reduced monocyte adhesion [331]. *Ripk1* knockdown in vivo in *Apoe*^*−/−*^ mice resulted in reduced atherosclerosis and inflammation, without affecting cholesterol levels, body weight, or liver enzymes, demonstrating that the effect is mediated explicitly by RIPK1 inhibition. Notably, RIPK1 inhibition did not impair the capacity of macrophages to undergo necroptosis [331]. Together, these findings identify RIPK1 as a key mediator of vascular inflammation and support its targeting in individuals at high risk of CAD [331].

#### RIPK2

RIPK2 is a component of the NOD-like receptor signaling pathways. It plays a significant role in regulating immune responses, including intracellular transport and modulation of immune responses, as well as in cancer development [355–357]. RIPK2 transmits signals by binding its CARD domain to NOD1 and NOD2, and it is subsequently ubiquitinated at multiple sites. This activates the NF-κB and MAPK signaling pathways, leading to inflammatory processes [358, 359]. In the absence of RIPK2, there is reduced production of pro-inflammatory cytokines such as TNF and IL-6, diminished neutrophil recruitment, and a weakened immune defense against intracellular pathogens [355, 360, 361]. In contrast, studies of atherosclerosis revealed that mice transplanted with *Ripk2*^*−/−*^ bone marrow developed more atherosclerotic lesions, despite a decreased local and systemic inflammatory response. Moreover, *Ripk2*-deficient macrophages unexpectedly took up more lipids despite reduced immune signaling, indicating that lipid accumulation was mediated through TLR4 rather than via scavenger receptors [333]. This paradoxical increase in lipid uptake was observed on multiple occasions. In an *in vivo ApoB100-Ldlr*^*−/−*^ bone-marrow transplanted model, *Ripk2*^*−/−*^ hematopoietic cells led to significantly enlarged lesions in the aorta and aortic root [333]. Furthermore, oil-red-O lipid uptake assays with minimally modified LDL, native LDL, and acetylated LDL revealed significantly increased lipid uptake in *Ripk2*^*−/−*^ macrophages compared to WT macrophages, which was confirmed by high-performance liquid chromatography (HPLC) analysis of triglycerides and cholesterol esters. Despite this enhanced lipid loading, NF-κB activity and pro-inflammatory cytokine production were reduced, consistent with the in vivo findings [333]. Knockdown of *Tlr4* expression abolished the increased lipid uptake in *Ripk2*^*−/−*^ macrophages, whereas knockdown of *Srb1* had no effect, indicating a TLR4-dependent mechanism. Further analysis revealed that RIPK2 normally suppresses TLR4/SYK-driven macropinocytosis; its absence leads to constitutive activation of this pathway, promoting LDL internalization and lipid accumulation [333]. Based on these results, RIPK2 is identified as a crucial regulator of macrophage lipid metabolism and is implicated in atherosclerosis and CVD [333].

### Atypical kinases

**Fig. 10:**
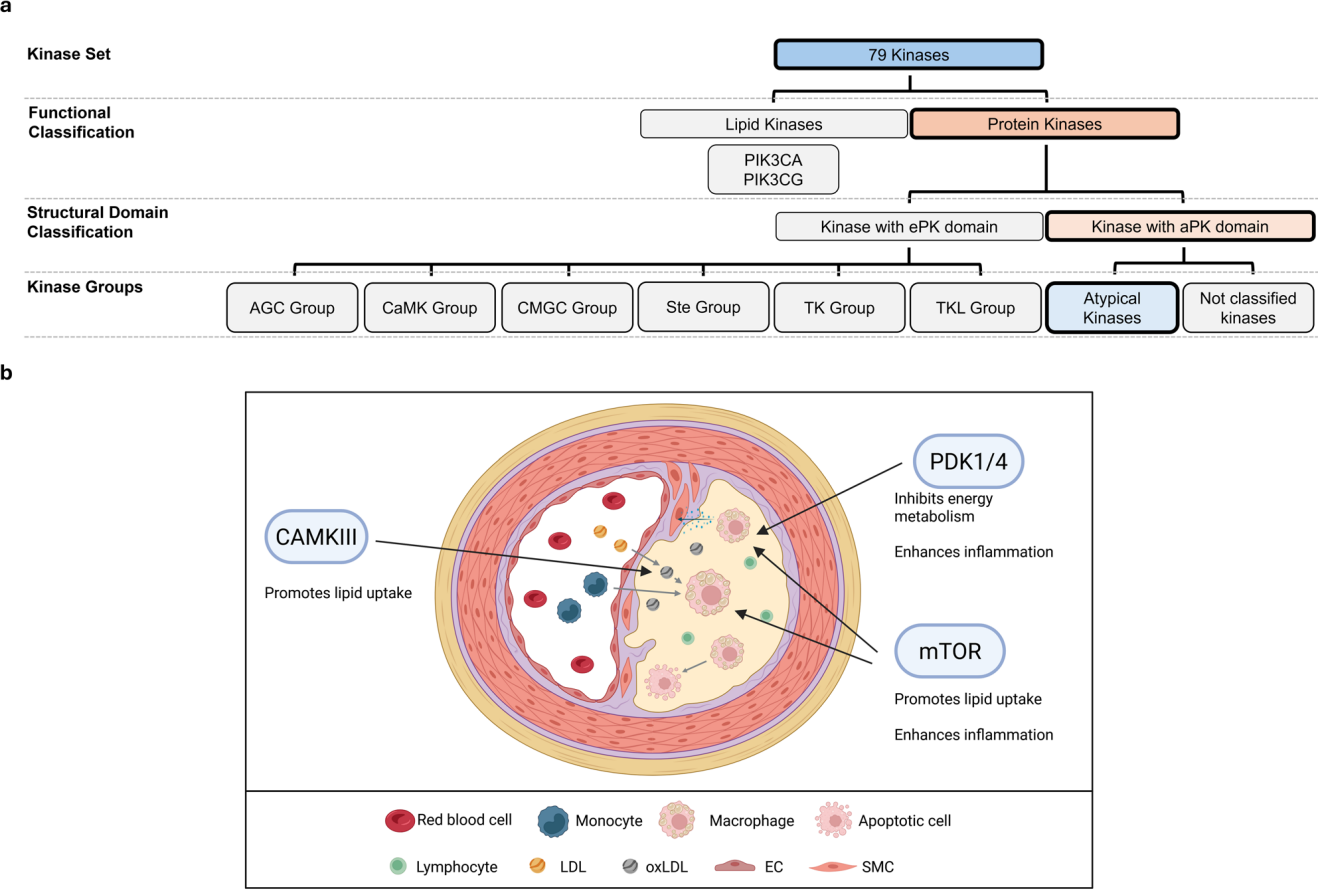
Overview of the role of the atypical kinases in atherosclerosis. **a** Classification of the 76 kinases into major structural and functional groups, highlighting the atypical kinases. **b.**Illustration of the cellular microenvironment of an atherosclerotic lesion and visualizing the involvement of atypical kinases. EC: endothelial cell; LDL: low-density lipoprotein; oxLDL: oxidized LDL; SMC: smooth muscle cell

In addition to the classical kinase groups, additional kinases largely lack the canonical ePK domain but share a common kinase-like structural fold, suggesting a shared evolutionary origin. However, to confirm a protein as an atypical kinase, functional validation beyond structural or sequence-based analysis is required [362]. Some atypical kinases have been implicated in atherosclerosis, including CaMKIII, mTOR, PIK3CA, PDK1, and PDK4 [363–367]. Additional kinases, such as ATM, ATR, TRPM7, and ULK1 can only be suggested to have relevant functions in atherosclerosis, as their involvement has not yet been fully characterized or experimentally validated [368–372] (Fig. 10).

#### CaMKIII

The atypical calcium-/calmodulin-dependent serine/threonine kinase eukaryotic elongation factor 2 kinase (CaMKIII, also known as Eef2k) is generally involved in the control of protein synthesis, cell survival, and cell proliferation. It is mainly activated under stress conditions such as nutrient deprivation or DNA damage [373, 374]. It influences cell survival by phosphorylating Eef2, thereby inhibiting its function [373]. As a result, the elongation phase of mRNA translation is slowed down, and the cell’s demand for energy and nutrients (particularly amino acids) is reduced. In addition, CaMKIII can modulate the expression of specific proteins by selectively interacting with particular mRNAs, thereby promoting polysome recruitment [363, 375–378]. In cancer research, CaMKIII has been extensively studied, and several functions are known, including supporting the migration of specific cancer cells and promoting the synthesis of pro-survival and pro-mitogenic proteins [363, 376, 379, 380]. However, less is known about the role of CaMKIII/Eef2k in inflammatory processes, particularly atherosclerosis. But some investigations examined its function in cholesterol-loaded macrophages and atherosclerotic models [363]. In oxLDL-treated BMDMs, Eef2k mRNA and protein activity were increased under cholesterol-loading conditions, while *Eef2k*-deficient macrophages formed fewer foam cells. Similarly, treatment of C57BL/6J BMDMs and human macrophages with the selective Eef2k inhibitor JAN-384 reduced foam cell formation. Across these models, Eef2k inhibition or knockout lowered CD36 protein expression, suggesting that CaMKIII/Eef2k promotes lipid uptake through CD36 [363]. In vivo, *Eef2k*^*−/−*^ *Ldlr*^*−/−*^ mice exhibited a reduced atherosclerotic burden compared with *Ldlr*^*−/−*^ controls. Consistent with the in vitro findings, plaque and blood analyses also demonstrated reduced CD36 expression in *Eef2k*-deficient conditions [363]. Human data further supported a role for Eef2k in atherosclerosis: PBMCs from patients with CAD and recent MI showed elevated Eef2k and CD36 mRNA expression compared with healthy controls [363]. Nevertheless, the contribution of CaMKIII/Eef2k to plaque stability remains unclear. Although *Eef2k* deficiency reduces foam cell formation and CD36 expression, the in vivo effects on plaque stability remain inconclusive. Thus, further investigation into CaMKIII/Eef2k-mediated regulation of inflammation in unstable plaques is warranted, to evaluate whether CAMKIII/Eef2k inhibition may represent a potential therapeutic strategy to lower atherosclerotic burden [363].

#### mTOR

The serine/threonine protein kinase mechanistic target of rapamycin (mTOR), a member of the phosphoinositol kinase-related kinase family (PIKK), regulates gene translation in response to nutrients, insulin, growth factors, and mitogens. It controls central cellular processes, including cell growth, differentiation, migration, and survival, and modulates immune cell activity and pro-inflammatory cytokine release during immune and inflammatory responses [381–384]. Consistently, mTOR signaling interacts with the NF-κB pathway to regulate monocyte adhesion and inflammatory activation, including via selectin-ligand and p-selectin glycoprotein ligand-1 (PSGL-1)-dependent AKT/mTOR/NF-κB-dependent signaling [385, 386]. mTOR inhibition, such as by rapamycin, exerts anti-proliferative effects and induces apoptosis or autophagic/lipophagic cell death in vitro and in vivo [387–397]. Therefore, the kinase became a target for cancer therapy and subsequently for CVDs. In atherosclerosis, mTOR inhibition can selectively deplete macrophages from plaques, improving plaque stability and reducing lesion burden [398–400]. Likewise, oxidative stress-induced inhibition of the PI3K/AKT/mTOR axis activates autophagy and promotes cholesterol efflux, contributing to atheroprotection [401–406]. Natural compounds, such as salvianolic acid B and oligomeric proanthocyanidins, also inhibit the PI3K/AKT/mTOR signaling, enhance autophagy, and reduce foam cell formation [407, 408]. Conversely, overactivation of mTOR by stimuli such as homocysteine, receptor ligands, or immune complexes suppresses autophagy and accelerates atherosclerosis [371, 372, 409, 410]. Furthermore, a lentivirus-mediated shRNA construct to inhibit mTOR (LV-shmTOR) was evaluated in *Apoe*^*−/−*^ mice. It has been shown that mTOR inhibition decreases total cholesterol, LDL cholesterol (LDL-C), and triglycerides, and increases HDL-C. In line with this, LV-shmTOR-treated *Apoe*^*−/−*^ mice showed reduced plaque size, increased fibrous cap thickness, and a higher cap-to-core ratio [365]. These results suggest that mTOR is a therapeutic target for stabilizing atherosclerotic plaques and thereby reducing atherosclerosis progression [365]. MiR-100, an upstream regulator of mTOR and raptor (components of mTOR complex 1 (mTORC1)), exhibits similar anti-inflammatory and anti-atherogenic effects, suggesting that an “anti-athero-miR” therapy could be a potential option for treating chronic vascular inflammation [411].

Further investigations shifted the focus to molecular mechanisms downstream of mTOR that regulate macrophage inflammation. This included energy metabolism, glycolysis, oxidative phosphorylation, and the role of prosaposin. Instead of viral gene silencing via LV-shmTOR, myeloid cell-specific nanobiologics containing either the mTOR inhibitor rapamycin (mTORi-NB) or the ribosomal protein S6 kinase beta-1 (S6K1) inhibitor PF-4,708,671 (S6K1i-NB) were used. Across mouse models, cell cultures, and human carotid plaques, the inhibition of mTOR and S6K1 reduced plaque inflammation, identifying a central role for prosaposin in immunometabolism [364]. mTOR/S6K1 inhibition in *Apoe*^*−/−*^ mice decreased plaque size, macrophage numbers, and protease activity, while S6K1i-NB only reduced macrophage numbers. Transcriptome analysis revealed prosaposin as a downregulated hub gene, with protein levels in plaques reduced by mTORi-NB and S6K1i-NB. Silencing prosaposin in BMDMs produced effects similar to those of mTOR/S6K1 inhibition, including suppressed glycolysis and oxidative phosphorylation. Consistently, loss of prosaposin in *Ldlr*^*−/−*^ mice reduced plaque volume and inflammatory cell content. Human monocyte and plaque analyses confirmed that prosaposin is highly expressed in macrophages, is regulated by oxLDL via the mTOR/S6K1 pathway, and is associated with pro-inflammatory plaque activity [364]. Due to these findings, prosaposin was identified as a key mediator of the anti-inflammatory effects downstream of mTOR/S6K1, highlighting it as a potential therapeutic target to limit atherosclerotic progression [364].

In addition to metabolic signals, psychological stress can also amplify mTOR-dependent inflammatory processes. Chronic stress altered the chromatin landscape and transcriptome of monocytes, transforming them into a primed, hyperinflammatory phenotype. RNA and assay for transposase-accessible chromatin (ATAC) sequencing of monocytes from the bone marrow of stressed and control mice, as well as of monocytes/hematopoietic cells from human blood, showed activation of the mTOR and PI3K metabolic pathways and reduced chromatin accessibility at loci associated with mitochondrial respiration. Moreover, these monocytes responded hyperresponsively to TLR ligands, exhibited a characteristic pro-inflammatory phenotype, and showed reduced mitochondrial respiration [412].

Further investigations sought an mTOR-activating factor derived from the daily diet, focusing on the association between high protein intake and atherosclerotic risk, mediated through the amino acid-mTORC1-autophagy signaling pathway, and the dose-response relationship between protein intake and this pathway in mice and humans. Downstream effects and amino acid specificity of mTORC1 activation were also evaluated. Notably, leucine, above a certain dietary threshold, activated mTORC1 in human monocytes/macrophages, thereby increasing the risk of atherosclerotic progression [366]. Analyses in overweight patients, supported by in vitro studies in human and murine macrophages, as well as validation in *Apoe*^*−/−*^ mice, confirmed the role of leucine in mTOR activation. High protein intake, compared with low protein intake, led to elevated plasma amino acids and mTORC1 activation shortly after a meal, along with decreased autophagy marker LC3. Increased colocalization of lysosome-associated membrane protein 2 (LAMP2) with mTOR was also observed under very high protein intake [366]. Amino acid profiling in patient plasma revealed that leucine, isoleucine, valine, methionine, threonine, serine, and arginine were elevated after high-protein meals. Among these, leucine showed the strongest activation of mTORC1, markedly higher than that of the other amino acids, and inhibited autophagy/mitophagy, increased ROS, and induced apoptosis [366]. In vivo results in *Apoe*^*−/−*^ mice confirmed a protein/leucine threshold that triggered an atherogenic effect. Increasing dietary protein produced a dose-dependent increase in mTOR activation in plaque macrophages, accompanied by increased plaque size, necrotic core area, macrophage numbers, and apoptosis [366]. Finally, a comparison of different diets with an iso-nitrogenous control (without additional leucine) revealed that leucine is the decisive trigger for mTORC1 activation in macrophages induced by high-protein intake [366]. These findings underscore the significance of daily food intake and the associated disease risks, exemplified by atherosclerosis, and the influence of amino acids on signaling processes, as shown for mTORC1 [366].

#### PDK1 and PDK4

The pyruvate dehydrogenase kinases 1–4 (PDK1-4) inhibit the activity of the mitochondrial pyruvate dehydrogenase (PDH), a major regulator of cellular energy metabolism in mammals, by phosphorylating specific serine residues [413, 414]. This reduces the conversion of pyruvate to acetyl coenzyme A (acetyl-CoA) and limits mitochondrial oxidative phosphorylation [415–417]. The four isoenzymes differ mainly in tissue distribution, but share up to 70% sequence homology [418–420]. PDK1 and PDK4 are central mediators of metabolic adaptation and inflammation, linking energy metabolism to immune cell activity. Their overexpression leads to decreased PDH activity, thereby promoting inflammatory processes, as shown for Alzheimer’s disease, multiple sclerosis, several types of cancer, and atherosclerosis [417, 421–425]. In atherosclerosis, the PDK/PDH axis, particularly the PDK1 isoenzyme, plays a central role. It was found that PDK1 and PDK4 expression in human carotid plaque tissue was positively correlated with the index of plaque vulnerability, and that the associated genes were enriched for immune-related pathways. Additionally, PDK4 showed higher expression than its isoforms. This suggests that higher levels of PDK1 and PDK4 are associated with unstable, inflammatory plaques [367]. Further investigations in a patient cohort revealed that high PDK1 expression was associated with significantly lower cardiovascular event-free survival. In contrast, no such association was found for the other PDK isoforms. Following proteomic analysis of human carotid tissue, PDK1 was the only isoenzyme upregulated in atherosclerotic plaques compared with healthy arterial tissue [367]. Furthermore, studies in human atheroma cells and *Apoe*^*−/−*^ mice have shown that the PDK/PDH axis can be targeted explicitly with DCA, resulting in reduced vascular inflammation and atherogenesis with minimal impact on body weight, cholesterol, and triglyceride levels [367]. At the immunological level in *Apoe*^*−/−*^ mice, DCA treatment led to a shift from M1 to M2 macrophages in the aorta and spleen, reduced pro-inflammatory T helper 1 (Th1) activity, inhibited NLRP3 activation in the vascular wall, and increased the regulatory T cell (Treg)/Th17 ratio in the spleen, with no noticeable effect on the liver. At the functional level, DCA inhibition in *Apoe*^*−/−*^ mice restored PDH function and reduced phosphorylation of PDK1. Additionally, PDK1 and PDK4 expression showed a trend toward correlation with aortic succinate levels, suggesting an association with macrophage-driven inflammation. Moreover, PDK1-specific knockdown in THP-1 macrophages resulted in a significant reduction in IL-1β secretion, implying that PDK1, rather than PDK4, controls inflammasome activation [367]. Because these findings highlight the pro-inflammatory influence of the PDK/PDH axis in atherosclerosis and specifically PDK1 as a key metabolic regulator of vascular inflammation, PDK1 inhibition may be a potential therapeutic approach for the secondary prevention of atherosclerosis. Therefore, further investigations with DCA or similar inhibitors should evaluate their efficacy, specificity, and safety to develop a new therapy that limits vascular inflammation and plaque progression [367].

### Not classified

**Fig. 11:**
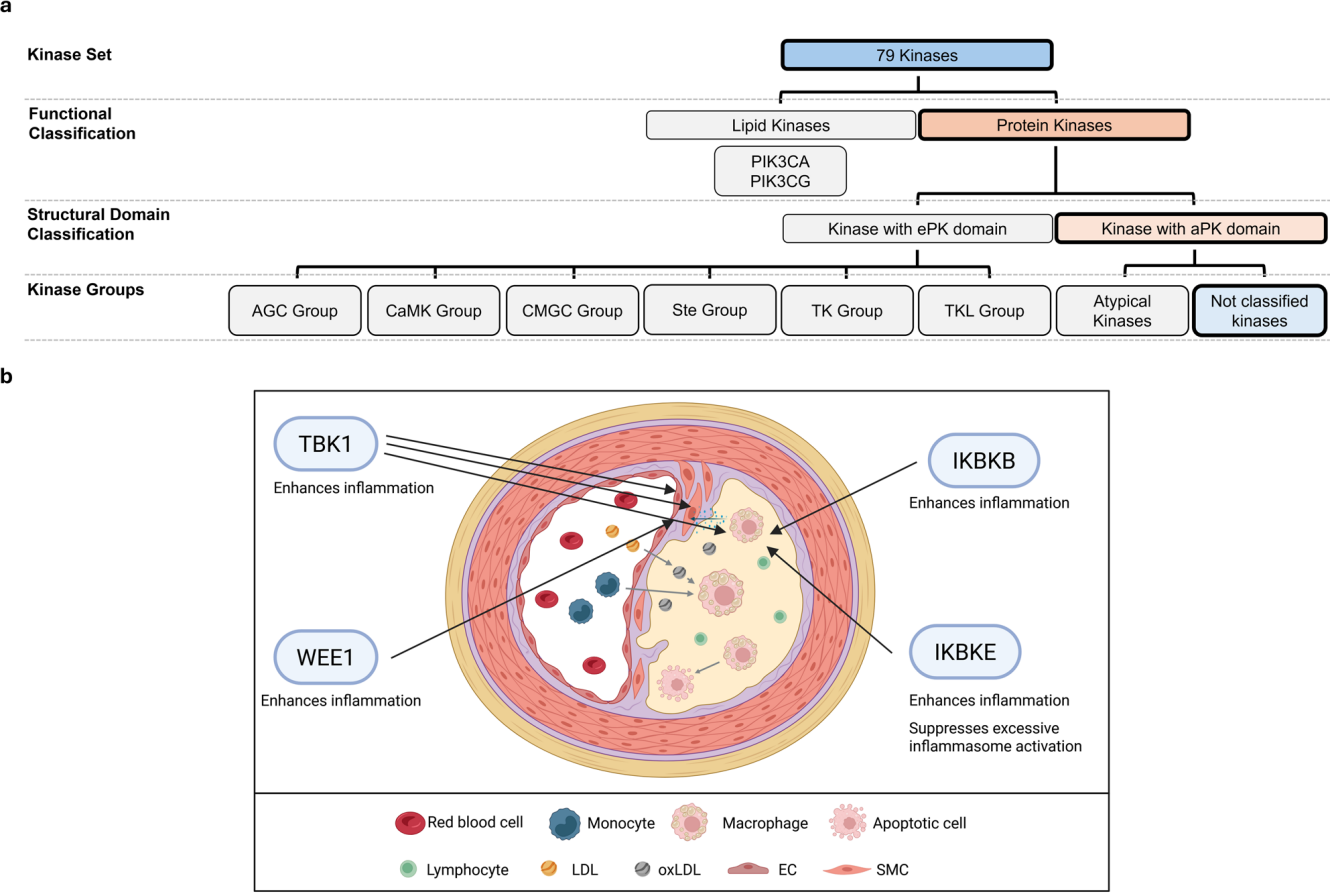
Overview of the role of the atypical kinases in atherosclerosis. **a** Classification of the 76 kinases into major structural and functional groups, highlighting the atypical kinases. **b** Illustration of the cellular microenvironment of an atherosclerotic lesion and visualizing the involvement of atypical kinases. EC: endothelial cell; LDL: low-density lipoprotein; oxLDL: oxidized LDL; SMC: smooth muscle cell

According to the classification system by Manning et al. [22], several protein kinases cannot be structurally assigned to any of the established kinase groups. Nonetheless, some of these unclassified kinases have been implicated in the pathophysiology of atherosclerosis, thereby justifying their classification as a distinct subgroup. Among these, IKBKB, IKBKE, TBK1, and WEE1 have explicitly been investigated in the context of atherosclerosis [328, 426–429]. At the same time, additional kinases, such as NEK7 and WNK1, have been proposed as potential contributors, although they have not yet been fully characterized functionally [28, 430–432] (Fig. 11).

#### IKBKB

The serine/threonine protein kinase IκB kinase β (IKKβ or IKBKB), together with IKKα and the NF-κB modulator (NEMO or IKKγ), forms the IκB kinase (IKK) complex [433]. IKBKB is the dominant kinase in the canonical NF-κB-dependent signaling pathway, where it mediates phosphorylation of IκB, leading to its degradation [434, 435]. This releases NF-κB, which then translocates to the nucleus to activate the expression of inflammatory genes. NF-κB is usually sequestered in the cytoplasm by IκB, and stimuli such as cytokines (e.g., TNF, IL-1) and bacterial components (e.g., LPS) trigger IKK-dependent activation of NF-κB-dependent signaling [434, 436, 437]. Inhibition of IKBKB, either directly with vinpocetine or indirectly with ebselen or MVP, can effectively suppress NF-κB-driven inflammation [436, 438]. Specifically, the intrinsic inhibitor MVP reduces IKBKB activation by binding to TRAF6 and impairing its oligomerization and polyubiquitination in macrophages. An MVP defect in *Apoe*^*−/−*^ mice resulted in enhanced NF-κB-dependent signaling, increased macrophage infiltration, and exacerbated obesity and atherosclerosis, thereby highlighting the role of IKBKB in CVD [328]. A close association was found between HDAC9 and the IKK complex in atherosclerosis, suggesting therapeutic potential for atheroprotection [426]. In *Apoe*^*−/−*^ mice on a WD, *Hdac9* deficiency resulted in smaller atherosclerotic lesions, reduced macrophage accumulation, a thicker fibrous cap, and a smaller necrotic core, thereby promoting a more stable plaque phenotype. Consistently, TMP195, a class IIa HDAC inhibitor, diminished endothelial activation and leukocyte-endothelial interactions. Consequently, pharmacological inhibition of HDAC9 reduced early lesion formation, leukocyte recruitment, and pro-inflammatory responses, demonstrating atheroprotective effects [426]. In mice with established lesions, TMP195 treatment further slowed disease progression, reduced monocyte and neutrophil infiltration, reduced necrotic core size, and increased fibrous cap thickness, thereby contributing to overall plaque stabilization [426]. Protein interaction analyses in HEK293 cells revealed that HDAC binds to IKKα and IKBKB, promoting their deacetylation and subsequent activation. This, in turn, enhanced NF-κB-dependent signaling and inflammatory responses. Consistent with this mechanism, RNA sequencing of TMP195-treated BMDMs showed that TMP195 downregulated key NF-κB-dependent inflammatory pathways, producing effects comparable to those observed upon IKBKB inhibition. Moreover, TMP195 reduced the production of inflammatory cytokines, such as IL-1β and IL-6, in ex vivo monocytes derived from patients with atherosclerosis [426]. Regarding inhibited central inflammatory pathways, reduced atherogenesis, and atheroprogression, these findings highlight the HDAC9 inhibitor TMP195 as a novel therapeutic strategy to alleviate vascular inflammation. Furthermore, they demonstrate that IKBKB mediates HDAC9-dependent inflammatory activity in atherosclerosis [426].

Another study, which specifically focused on the kinase DCLK1, likewise identified a direct interaction between DCLK1 and IKBKB. As noted previously, DCLK1 directly binds to and activates IKBKB. Genetic deletion or pharmacological inhibition of DCLK1 with the DCLK1-IN-1 inhibitor in *Apoe*^*−/−*^ mice attenuated atherosclerosis and vascular inflammation and reduced immune cell infiltration. Moreover, *Ikbkb* knockdown blocked DCLK1-mediated NF-κB activation. Thus, it was emphasized that, in addition to DCLK1, IKBKB is a relevant therapeutic target for the treatment of atherosclerosis [58].

#### IKBKE

The serine/threonine protein kinase inhibitor of κB kinase epsilon (IKBKE) plays a central role in regulating inflammatory pathways and mediating type I antiviral immune responses [439]. Activation of these antiviral mechanisms is initiated by pattern recognition receptors and their associated signaling adaptors, including TLR3 and TLR4 via TIR-domain-containing adapter-inducing interferon-β (TRIF), as well as the mitochondrial antiviral signaling protein (MAVS) signaling cascade [440, 441]. IKBKE expression is predominantly restricted to immune cells, where it acts as a downstream effector of TLR signaling and is transcriptionally regulated by NF-κB [428]. In addition, oncogenic stimuli and obesity have been shown to induce dysregulated IKBKE expression in mice [442–444]. However, experimental findings regarding the influence of IKBKE on body weight regulation have been inconsistent [428, 445]. Further studies have highlighted the role of IKBKE in atherosclerosis, showing the positive correlation between IKBKE mRNA expression levels and body mass index (BMI) in human adipose tissue. BMDMs from *Apoe*^*−/−*^ mice strongly expressed IKBKE in response to pro-inflammatory signals. In contrast, anti-inflammatory stimuli, such as IL-4, suppressed its expression, indicating a cell-autonomous role in regulating chronic inflammation [428]. *Ikbke* knockout in M1 macrophages led to elevated NLRP3 expression and enhanced IL-1β secretion, suggesting that IKBKE constrains the magnitude and duration of inflammasome activation. These findings were confirmed in vivo in *Ikbke*-deficient *Apoe*^*−/−*^ mice, which exhibited higher expression of inflammasome-related genes, increased macrophage markers, and enlarged fat depots. In contrast, dKO *Apoe*^*−/−*^ *Ikbke*^*−/−*^ mice transplanted with bone marrow from WT *Apoe*^*−/−*^ donors displayed reduced inflammasome gene expression and pro-inflammatory markers, decreased fat depots, fewer and smaller adipocytes, lower leptin and insulin levels, and improved cholesterol clearance. Despite these beneficial effects on metaflammation, global *Ikbke* deficiency did not affect overall plaque size but did alter its composition. An increased number of terminal deoxynucleotidyl transferase-mediated dUTP nick-end labeling (TUNEL)-positive cells and reduced smooth muscle actin (SMA^+^) areas were observed in mice with hematopoietic IKBKE expression [428]. These results indicate that IKBKE expression in hematopoietic cells is sufficient to mitigate excessive inflammatory and adipose tissue alterations, but has less influence on atherosclerosis progression [428].

#### TBK1

TBK1 is a serine/threonine protein kinase that belongs to the noncanonical IKK family, together with IKKε [446]. TBK1 is ubiquitously expressed and has cell-specific functions that have been characterized in mouse models, including monocytes and macrophages. In murine macrophages, TBK1 is involved in TLR3- and TLR4-dependent signaling [441] and regulates NF-κB-dependent inflammatory responses [447–449] and IRF-dependent interferon signaling [450–452]. Elevated TBK1 activity has been observed in inflammatory disease models, including metabolic and vascular disease-related contexts, and is associated with chronic inflammation [446]. Further studies in *Ldlr*^*⁻/⁻*^ mouse models, MOVAS cells, and human cell systems, including HAoECs and THP-1 monocytes, assessed the effects of TBK1/IKBKE inhibition using the dual inhibitor amlexanox [444]. Amlexanox-treated *Ldlr*^*⁻/⁻*^ mice exhibited a reduced aortic lesion area with reduced macrophage accumulation and significantly smaller plaques in the aortic root, indicating an anti-atherogenic effect of amlexanox and supporting a role of TBK1/IKBKE-regulated pathways in lesion formation [429]. Moreover, oral gavage with ¹⁴C-cholesterol resulted in decreased serum radioactivity accompanied by increased radioactivity in the liver, bile, and feces. This finding indicates that inhibition of TBK1/IKBKE enhances cholesterol clearance, with the effect occurring via increased excretion rather than altered absorption or synthesis. In addition, liver RNA-sequencing analysis revealed upregulation of bile acid synthesis and metabolism pathways, along with increased expression of cholesterol 7α-hydroxylase (Cyp7a1), the rate-limiting enzyme in bile acid synthesis. Consistently, quantification of fecal bile acid content demonstrated significantly elevated levels, confirming a functional role of TBK1/IKBKE inhibition in stimulating bile acid-mediated cholesterol excretion. Further aortic RNA-sequencing analysis of *Ldlr*^*⁻/⁻*^ mice revealed downregulation of inflammatory pathways, reduced TNF-α and TGF-β signaling, and suppression of SMC proliferation and migration programs [429]. Therefore, inhibition of TBK1 and IKBKE may represent a potential therapeutic target for the treatment of atherosclerosis and hypercholesterolemia that should be further explored in future studies [429].

#### WEE1

The serine/threonine kinase WEE1 plays a crucial role in cell cycle regulation, particularly at the G2/M checkpoint. There, WEE1 phosphorylates CDK1 at tyrosine 15, preventing premature mitotic entry, allowing time for DNA repair, and maintaining the stability of the replication fork [453, 454]. In tumors, WEE1 overexpression compensates for elevated replication stress, making it a key kinase in cancer progression [455, 456]. Further studies identified WEE1 as a key regulator in atherosclerosis, acting upstream of p65. *Apoe*^*−/−*^ mouse models and human carotid tissue showed elevated WEE1 activity, with unchanged protein levels [427]. Its activation was induced by Ser642 phosphorylation in *Apoe*^*−/−*^ mice and was predominantly observed in macrophages within atherosclerotic plaques. These findings were confirmed in oxLDL-stimulated MOVAS, HUVECs, and mouse peritoneal macrophages (MPMs), with only MPMs showing a time-dependent increase in p-WEE1 (Ser642). Additionally, *Wee1* knockout in oxLDL-stimulated MPMs predominantly altered genes related to inflammatory response and immune system processes by inhibiting oxLDL-induced upregulation of inflammatory genes, such as TNF and IL-6. In contrast, WEE1 overexpression elevated and exacerbated cytokine secretion in oxLDL-stimulated MPMs. The same effect was achieved by self-activation of WEE1, without oxLDL stimulation, demonstrating that kinase-dependent activation of WEE1 is sufficient to induce inflammation. Pharmacologic inhibition with MK1775 in MPMs confirmed this observation, as it dose-dependently inhibited oxLDL-induced cytokine secretion [427]. Furthermore, the pharmacological inhibition of WEE1 decreased atherosclerotic plaque progression and increased plaque stability, without affecting the serum lipid profile, as demonstrated in *Apoe*^*−/−*^ mice that underwent bone marrow transplantation from WEE1^f/f^ or WEE1^MCKO^ donors (genetic inhibition of WEE1 in macrophages) [427]. The *Apoe*^*−/−*^WEE1^MCKO^ mice exhibited reduced aortic plaque size and increased plaque collagen content, contributing to plaque stability. Moreover, macrophage, neutrophil, and monocyte infiltration was reduced in atherosclerotic lesions, accompanied by lower systemic and local inflammatory responses. These results were further confirmed in *Apoe*^*−/−*^ mice treated with the pharmacologic WEE1 inhibitor MK1775. Reduced atherosclerosis, plaque stabilization, and attenuation of inflammatory cell infiltration and systemic and local inflammatory responses were again demonstrated, without affecting lipid profiles. The underlying signaling pathway involves NF-κB, which is activated by phosphorylation of the transcription factor p65 at Ser536 by WEE1. Activated p65 translocates into the nucleus and promotes the expression of pro-inflammatory cytokines. *Wee1*-knockout, therefore, suppresses p65 activation and reduces p65 phosphorylation in plaques, indicating the central role of this mechanism in macrophage-mediated inflammation and plaque progression in atherosclerosis [427]. The specific binding of WEE1 to p65 was further confirmed by 293T-cells transfected with hemagglutinin tag (HA)-WEE1 plasmids. This complex was identified in both oxLDL-treated macrophages and in atherosclerotic lesions of *Apoe*^*−/−*^ mice, and its activation depended on WEE1 phosphorylation [427]. By effectively inhibiting the WEE1/p65 complex, either genetically through macrophage-specific deletion or pharmacologically with the selective kinase inhibitor MK1775, NF-κB-dependent inflammation and atherosclerotic progression can be suppressed. This highlights WEE1 as a potential therapeutic target for atherosclerosis.

### Lipid kinases

In addition to protein kinases, the literature search criteria for this systematic review identified two lipid kinases, phosphatidylinositol-4,5-bisphosphate 3-kinase catalytic subunit alpha (PIK3CA) and PIK3CG, which are associated with atherosclerosis. However, they were not directly described, indicating a potential target that remains open to investigation into atherosclerosis [384, 406, 457].

## Conclusions

Investigations into the roles of kinases in macrophages in atherosclerosis provide initial insights into the underlying complexity of the signaling network and offer different approaches for developing new therapeutic strategies. Still, only a fraction of the 509 kinases have been closely investigated in atherosclerosis, and further research in this field is required. Moreover, understanding the impact of inhibiting or promoting one kinase on atherosclerotic symptoms, such as plaque formation, collagen deposition, cholesterol accumulation, or the recruitment of macrophages, as well as on subsequent components of a signaling cascade, like NF-κB, facilitates an understanding of the fundamental processes involved in atherosclerosis and offers potential targets for specific drugs or similar treatments.

Additional knowledge from cancer research and other inflammatory diseases can be applied to CVD by transferring established strategies, such as testing the same drugs in atherosclerotic mouse models or in human cell cultures, or by employing similar approaches. Furthermore, metabolic parameters that have been shown to affect atherosclerosis development, such as nutrition or psychological stress, may also provide essential information about kinase activity and their pro- or anti-inflammatory responses. Especially lifestyle-related factors with high impact on atherosclerotic progression are particularly relevant for society, as atherosclerosis is a silent inflammation until stroke or MI occurs. Nevertheless, to prevent or reduce the atherosclerotic burden, further research on specific kinase inhibitors or modulators is necessary, as is mapping the remaining kinases across cell types, including ECs, SMCs, and T cells.

In conclusion, this systematic review provides broad insight into the current state of research on macrophage kinases in atherosclerosis (Table 2). Still, it represents a fragment of current knowledge of atherosclerotic processes and highlights the multifaceted, multilayered nature of the disease, which demands broader and deeper investigation of kinase-mediated mechanisms.

**Table 2:** Summary table of highly relevant manuscripts

Kinase	Prim./ Sec. effects	Main signaling pathway	Mouse model	Human evidence	Inhibitors/ modulators	Ref.
Akt1	Sec.	PI3K-Akt1-eNOS in ECs	*Apoe* ^*−/−*^ *Akt1* ^*−/−*^	None	None	[26]
Akt1	Prim.	PI3K-Akt1-GSK3	*Apoe* ^*−/−*^ *Sr-b1* ^*−/−*^ *Apoe* ^*−/−*^ *Sr-b1* ^*−/−*^ *Akt1* ^*−/−*^	None	CD36-blocking peptides	[25]
ALK1 (ACVRL1)	Sec.	TGF-β / BMP-ALK1-SMAD1/5/8	*Ldlr* ^*‑/‑*^ mice PCSK9‑over-expression	↑ ALK1 in human atherosclerotic arteries	EC-specific *Alk1* deletion mAb2 blocking LDL binding/uptake/trans-cytosis	[327]
BTK	Prim.	BTK-NF-κB	*Apoe* ^*−/−*^ ± sh-BTK	None	BTK-specific siRNA	[166]
BTK	Prim.	BTK-NLRP3-caspase 1-IL-1β	*Apoe* ^*−/−*^	None	Ibrutinib encapsulated in a polydopamine-PEG nanoparticle	[167]
CaMK4	Prim.	Ca^2+^-CaMKK2-CaMK4-ATF6 / CREB	PCSK9‑over-expression	↑ CaMK4 in unstable carotid plaques	KN-93 inhibitor Inactive analog KN-92 CaMK4-specific siRNA	[59]
CaMKIII (Eef2k)	Prim.	Ca^2+^ -calmodulin-Eef2k-Eef2	*Ldlr* ^*−/−*^ *Eef2k* ^*−/−*^ *Ldlr* ^*−/−*^	↑ Eef2k in PBMCs from CAD patients	Small-molecule Eef2k inhibitor JAN-384	[363]
DCLK1/ IKBKB	Prim.	DCLK1-IKKβ- NF-κB	*Apoe* ^*−/−*^ *Lyz2* ^*cre*^ *Dclk1* ^fl/fl^	None	Small molecule DCLK1 inhibitor DCLK1-IN-1	[58]
EGFR	Prim.	TLR4-c-Src-EGFR-Akt/ERK-NF-κB	*Apoe* ^*−/−*^	None	Small-molecule EGFR inhibitor AG1478 EGFR inhibitor compound 542	[169]
EphA2	Prim.	HDAC11-KLF4-EphA2-NLRP3-NF-κB	*Apoe* ^*−/−*^	None	EphA2 inhibitor ALW-II-4127 Adenoviral siEphA2	[170]
EphA2	Prim.	Involves suppression of NF-κB and NLRP3	*Apoe* ^*−/−*^	↑ EphA2 in PBMCs from CAD patients EphA2 is inversely linked to the bile acid profile	ALW-II-4127	[239]
FES	Prim.	PRDM1-chromatin-FES	*Apoe* ^*−/−*^ *Fes* ^*−/−*^	rs17514846 ◊ ↓ FES (monocytes/SMCs) Low macrophage FES ◊ ↑ plaque burden	CRISPR editing of rs17514846 and rs1894401 FES-specific siRNA	[171]
FGFR1	Prim.	FGFR1-PLCγ-NF-κB	*Apoe* ^*−/−*^ *Apoe* ^*−/−*^ *Fgfr1* ^*−/−*^	↑ FGFR1 in coronary plaques; Enriched in CD68⁺ macrophages	FGFR1-4 inhibitor AZD4547	[172]
IKBKB	Prim.	Canonical NF-κB pathway centered on HDAC9-driven IKBKB activation	*Apoe* ^*−/−*^ *Hdac9* ^*−/−*^ Bone marrow chimeras	TMP195 ◊ ↓ IL-1β / IL-6 in human monocytes	Selective class IIa HDAC inhibitor TMP195 Small-molecule IKBKB inhibitor TPCA-1	[426]
IKBKE	Prim.	TLR-NF-κB-NLRP3	*Apoe* ^*−/−*^ *Ikbke* ^*−/−*^ Bone marrow chimeras	None	None	[428]
MAP4K4	Prim.	LPS-TLR4-MAP4K4	*Apoe* ^*−/−*^	None	MAP4K4-specific siRNA	[138]
MAPK1	Prim.	RAF-MEK-MAPK	*Jnk1* ^*−/−*^	None	MEK1/2 inhibitor U0126 and PD98059	[103]
MAPK8 (JNK1)	n.s.	JNK1-RIPK1-caspase-3	*Ldlr* ^*−/−*^	None	JNK‑Inhibitor CT536706 JNK1-specific siRNA	[102]
MAPK8 (JNK1)	n.s.	JNK1-caspase-3	*Ldlr* ^*−/−*^ reconstituted with *Jnk1*^*−/−*^ or *Jnk2*^*−/−*^ bone marrow	None	None	[100]
MAPK8 (JNK1)	Prim.	JNK1-PTEN-Akt/Bad-apoptosis	C57BL/6	None	Anisomycin Selective JNK1-inhibitor JNKI1	[101]
MAPK8 (JNK1)	Prim.	JNK1/CD14/SR-AI	C57BL/6 *Jnk2* ^*−/−*^	None	Unspecific JNK inhibitor SP600125 MEK1/2 inhibitor U0126	[130]
MAPK9 (JNK2)	n.s.	JNK2/ROS/NO	*Apoe* ^*−/−*^	None	None	[105]
MAPK9 (JNK2)	Prim.	JNK2/NF-κB/STAT3	*Apoe* ^*−/−*^	None	Peptide-siRNA nanostructures (p5RHH-JNK2 siRNA)	[106]
MerTK	Prim.	No primary focus	*Apoe* ^*−/−*^	None	Hybrid membrane nanovesicles (HMNVs) expressing MerTK	[177]
mTOR	Prim.	mTOR	*Apoe* ^*−/−*^ *Ldlr* ^*−/−*^	None	Lentivirus-mediated shRNA LV-shmTOR	[365]
mTOR	Prim.	mTOR/S6K1	*Apoe* ^*−/−*^ *Ldlr* ^*−/−*^	↑ PSAP-mTOR in plaque macrophages	Rapamycin, PF-4,708,671 and Prosaposin	[364]
PDK1	Prim.	PDK/PDH	*Apoe* ^*−/−*^	↑ PDK1/PDK4 in carotid plaques High plaque PDK1 ◊ ↓event-free survival	Dichloroacetat	[367]
PRKCD	Prim.	PKCδ-PI3K/Akt-ERK-PKCβ	None	↑ PKCδ/AKT/ p-ERK/SR-A in plaque macrophages	PKCδ-inhibitor rottlerin	[24]
RIPK1	Prim.	RIPK1-TNF-IKK-NF-κB	*Apoe* ^*−/−*^	↑ RIPK1 in human plaques	None	[331]
RIPK2	Prim.	TLR4/SYK	*ApoB100-Ldlr* ^*−/−*^ reconstituted with *Ripk2* ^*−/−*^ bone marrow	None	None	[333]
Stk25	Sec.	No primary focus	PCSK9‑over-expression	None	None	[164]
SYK	Sec.	No primary focus	*Ldlr* ^*−/−*^	None	Fostamatinib and R406	[179]
SYK	Prim.	SYK/Kmt5a/H4K20me (Epigenetic pathway)	*Apoe* ^*−/−*^	↑ SYK/KMT5A in monocytes from STEMI patients SYK correlates with plaque burden (Gensini)	Histone methyltransferase inhibitor UNC0379	[180]
TBK1	Sec.	TBK1/IKBKE	*Ldlr* ^*−/−*^	None	Amlexanox	[429]
Trib1	Prim.	Trib1/Olr1	*Apoe* ^*−/−*^ PCSK9‑over-expression	None	None	[57]
WEE1	Prim.	WEE1/NF-κB	*Apoe* ^*−/−*^	↑ WEE1 in carotid plaque macrophages	MK1775	[427]

*N.s*. Not specified, *Prim*. Primary, *Ref*. Reference, *Sec*. Secundary

## Data Availability

No datasets were generated or analysed during the current study.

## Abbreviations

6-OHDA: Neurotoxin 6-hydroxydopamine
AAV-PCSK9: Adeno-associated virus expressing proprotein convertase subtilisin/kexin type 9
ABCA1: ATP-binding cassette transporter A1
ABCG1: ATP-binding cassette subfamily G member 1
ACAT: Acyl-CoA: cholesterol acyltransferase
ACE: Angiotensin-converting enzyme
acetyl-CoA: Acetyl coenzyme A
ACK: Activated Cdc42-associated kinase
Acvrl1: Activin-like kinase receptor 1
ADAM10: A disintegrin and metalloproteinase 10
AGC: Protein kinase A/G/C related group
AML12: Alpha mouse liver 12
AMPK: Adenosine monophosphate-activated protein kinase
AnxA2: Annexin A2
AP-1: Activator protein 1
APJ: Apelin receptor
Apoe: Apolipoprotein E
Arg1: Arginase 1
ASK1: Apoptosis signal-regulating kinase 1
ASXL1: Additional sex combs-like 1
ATAC: Assay for transposase-accessible chromatin
Atf6: Activating transcription factor 6
Atg13: Autophagy-related protein 13
ATM: Ataxia-telangiectasia mutated
ATP: Adenosine triphosphate
ATR: Ataxia-telangiectasia and Rad3-related
BCL10: B-cell lymphoma/leukemia 10 protein
BCR: B-cell receptor
BMDMs: Bone marrow-derived macrophages
BMI: Body mass index
BMMCs: Bone marrow-derived mononuclear cells
BMP9: Bone morphogenetic protein-9
Bmx-Cre-ERT2: Bone marrow tyrosine kinase on chromosome X cre-recombinase estrogen receptor 2
BTK: Non-receptor-associated Bruton’s tyrosine kinase
c-fms: Cellular feline McDonough Sarcoma oncogene
c-Src: Cellular sarcoma
Ca2+: Calcium
CAB39: Calcium-binding protein 39
CABG: Coronary artery bypass grafting
CAD: Coronary artery disease
CaM: Calmodulin
CaMK: Calcium/calmodulin-dependent protein kinase
CaMK2γ: Calcium/calmodulin-dependent protein kinase type II gamma
CaMK4: Calcium/calmodulin-dependent protein kinase IV
CaMKK2: Calcium/calmodulin-dependent protein kinase kinase 2
CARD: Caspase recruitment domain protein
CCL2: C-C motif chemokine ligand 2
CCR2: C-C chemokine receptor type 2
CD36: Cluster of differentiation 36
CDKs: Cyclin-dependent kinases
CEA: Carotid endarterectomy
CEL: Nε-carboxyethyl-lysine
CH: cConal hematopoiesis
ChIP: Chromatin Immunoprecipitation
CLKs: CDK-like kinases
CMGC: Cdk, MAPK, GSK, Cdk-like related group
CNBP: CCHC-Type Zinc Finger Nucleic Acid Binding Protein
COX-2: Cyclooxygenase-2
CREB: cAMP response element-binding protein
CRP: C-reactive protein
CSF-1R: Colony-stimulating factor 1 receptor
CVD: Cardiovascular disease
CX3CR1: C-X3-C motif chemokine receptor 1
CXCL11: C-X-C motif chemokine ligand 11
Cyp7a1: Cholesterol 7α-hydroxylase
DAG: Diacylglycerol
DAMPs: Damage-associated molecular patterns
DCA: Deoxycholic acid
DCLK1: Doublecortin-like kinase 1
DCLK1-IN-1: DCLK1-inhibitor-1
dKO: Double knockout
DNMT3A: DNA (cytosine-5)-methyltransferase 3 A
DOPE: 1,2-dioleoyl-sn-glycero-3-phosphoethanolamin
DRP1: Dynamin-related protein 1
EBPβ: Enhancer binding protein β
ECs: Endothelial cells
Eef2k: Eukaryotic elongation factor 2 kinase
EGFR: Epidermal growth factor receptor
eLDL: Enzymatically modified low-density lipoproteins
eNOS: Endothelial nitric oxide synthase
EphA2: Ephrin type-A receptor 2
ePK: Eukaryotic protein kinase
eQTL: Expression quantitative trait locus
ER: Endoplasmic reticulum
ERK: Extracellular signal-regulated kinase
ESC: Embryonic stem cell
FAK: Focal adhesion kinase
Fc: Fragment crystallizable
FER: Feline erythroblastosis virus-associated receptor
FES: Feline sarcoma oncogene
FGF1: Fibroblast growth factor 1
FGFR1: Fibroblast growth factor receptor 1
FIS1: Fission 1 protein
FPS: Feline sarcoma proto-oncogene
FRS2: Fibroblast growth factor receptor substrate 2
Gas6: Growth arrest-specific 6
GCK-IV: Germinal cell kinase four
GeRPs: β1,3-d-glucan-encapsulated siRNA particles
GFP: Green fluorescent protein
GOLM1: Golgi membrane protein 1
GPI: Glycosylphosphatidylinositol
GpVI: Glycoprotein VI
Gr-1: Granulocyte marker 1
GSKs: gGlycogen synthase kinases
GTPase: Guanosin-triphosphatase
HA: Hemagglutinin tag
HA-VSMC: Human aortic vascular SMC
HAoEC: Human aortic endothelial cell
HBMEC: Human brain microvascular endothelial cell
HCAEC: Human coronary artery endothelial cell
HCD: High-cholesterol diet
HCV: Hepatitis C virus infection
HDAC11: Histone acetylase 11
HDL: High-density lipoprotein
HEK293T cells: Human embryonic kidney 293 T cells
HepG2: Human epithelioma G2
HMDP: Highly multiplexed digital profiling
HMGCR: 3-hydroxy-3-methylglutaryl-CoA reductase
HMNV: Hybrid membrane nanovesicle
HPLC: High-performance liquid chromatography
HSCs: Hematopoietic stem cells
HUVEC: Human umbilical vein endothelial cell
ICAM-1: Intercellular adhesion molecule-1
IFN-γ: Interferon-gamma
IHC: Immunohistchemical analysis
IKBKE: Inhibitor of κB kinase epsilon
IKK: IκB kinase
IKKβ: IκB kinase β
IL-6: Interleukin-6
IL-6R: IL-6 receptor
Il1r1-/-: IL-1 receptor 1-deficient
iNos: Inducible nitric oxide synthase
IP-10: Interferon-γ-induced protein-10
iPSC: Induced pluripotent stem cell
IRAK: IL-1 receptor-associated kinase
IRE1: Inositol-requiring enzyme 1
IRFs: Interferon regulatory factor
ITCH: Itchy E3 ubiquitin protein ligase
JAK2: Janus kinase 2
JAK2VF: JAK2V617F
JNK: C-jun N-terminal kinase
Kg: Kilogram
KI: Knock-in
KLF4: Krüppel-like factor 4
Kmt5a: Lysine methyltransferase 5 A
KN93: N-[2-[[[3-(4-Chlorophenyl)-2-propenyl]methylamino]methyl]phenyl]-N-(2-hydroxyethyl)-4-methoxybenzenesulfonamide
LAB: Low allele burden
LAMP2: Lysosome-associated membrane protein 2
LC3-II: Lipidated form of microtubule-associated protein 1 light chain 3
Lcn2: Lipocalin 2
LDL: Low-density-lipoprotein
LDL-C: LDL-cholesterol
LDLr: LDL receptor
LPL: Lipoprotein lipase
LPS: Lipopolysaccharide
LV-shmTOR: Lentivirus-mediated shRNA construct
LXR: Liver X receptor
Ly6C: Lymphocyte antigen 6 complex, locus C
Lyn: Lck/Yes-related novel tyrosine kinase
LysM: Lysozyme-C
M-CSF: Macrophage-CSF
mAb: Monoclonal antibody
MALT1: Mucosa-associated lymphoid tissue lymphoma translocation protein 1
MAPK: Mitogen-activated protein kinase
MKP1: MAP kinase phosphatase 1 (MKP1)
MAVS: Mitochondrial antiviral signaling protein
MCP-1: Monocyte chemoattractant protein-1
MDM: Monocyte-derived macrophage
MEK: MAPK/ERK kinase
MEKK: MAPK/ERK kinase kinase
MerTK: Myeloid-epithelial-reproductive tyrosine kinase
MI: Myocardial infarction
MiR: MicroRNA
MKK: MAP kinase kinase
MKKK: MAP kinase kinase kinases
MLEC: Mouse lung endothelial cell
MLK: Mixed-lineage kinase
MMP-9: Matrix metalloproteinase-9
MPM: Mouse peritoneal macrophages
MPN: Myeloproliferative neoplasms
mRNA: Messenger ribonucleic acid
mTmG: Membrane-targeted tomato membrane-targeted GFP
mTOR: Mechanistic target of rapamycin
mTORC1: mTOR complex 1
mTORi-NB: mTOR inhibitor rapamycin
mTrib1: Myeloid-specific Trib1
MVP: Major vault protein
MyD88: Myeloid differentiation primary response 88
NADPH: Nicotinamide adenine dinucleotide phosphate
NEAT1: Nuclear-enriched abundant transcript 1
NEMO: NF-κB modulator
NET: Neutrophil extracellular trap
NF-κB: Nuclear factor kappa-light-chain-enhancer of activated B cells
NLRP3: Nucleotide-binding oligomerization domain-like receptor family, pyrin domain-containing protein 3
NOD: Nucleotide-binding oligomerization domain
Nr4a1: Nuclear receptor subfamily 4, group A, member 1
NRF2: Nuclear factor erythroid 2-related factor 2
Olr1: Oxidized low-density lipoprotein receptor 1
oxLDL: Oxidized low-density lipoprotein
p130Cas-Crk: CT10 regulator of kinase-associated substrate
PBMC: Peripheral blood mononuclear cell
PDHc: Pyruvate dehydrogenase complex
PDK1-4: Pyruvate dehydrogenase kinases 1–4
PEG-SOD: Polyethylene glycol-superoxide dismutase
PERK: Protein kinase R-like ER kinase
PI3K: Phosphoinositide 3-kinase
PIK3CA: Phosphatidylinositol-4,5-bisphosphate 3-kinase catalytic subunit alpha
PIKK: Phosphoinositol kinase-related kinase family
PIP-CD47: CD47-targeted polydopamine-based nanocarrier system
PKC: Protein kinase C
PLCγ: Phospholipase C gamma
PP2: 4-amino-5-(4-chlorophenyl)-7-(t-butyl)pyrazolo[3,4-d]pyrimidine
PRDM1: Positive regulatory domain I-binding factor 1
PSGL-1: P-selectin glycoprotein ligand-1
Ptges: Prostaglandin E-synthase
PTK2: Protein tyrosine kinase 2
Pyk2: Proline-rich tyrosine kinase 2
qPCR: Quantitative polymerase chain reaction
RACE: 5’ rapid amplification of cDNA ends
RAF: Rapidly accelerated fibrosarcoma kinase
RAGE: Receptor for advanced glycation end products
RAS: Rat sarcoma virus-related small GTPase
RhoBTB: Rho-related BTB-domain containing protein
RIPK1: Receptor-interacting protein kinase 1
RNA: Ribonucleic acid
RNAi: RNA interference
ROS: Reactive oxygen species
RTK: Receptor tyrosine kinase
RvD1: Resolving D1
S6K1: Ribosomal protein S6 kinase beta-1
S6K1i-NB: S6K1 inhibitor PF-4708671
SBA: Secondary bile acid
SCFA: Short-chain fatty acid
SH2: Src homology 2
shRNA: Short hairpin RNA
siRNA: Small interfering RNA
SIRT1: Sirtuin 1
SMA: Smooth muscle actin
SMC: Smooth muscle cell
SMN: Superparamagnetic iron oxide nanoparticles
SNP: Single-nucleotide polymorphism
SNX10: Sorting nexin 10
SR: Scavenger-receptor
Srb1: Scavenger-receptor class B type I
STAT3: Signal transducer and activator of transcription 3
STEMI: ST-elevation myocardial infarction
Stk25: Serine/threonine kinase 25
STRAD: STE20-related adaptor
SYK: Spleen tyrosine kinase
TAK1: Transforming growth factor-β-activated kinase
TAK242: Ethyl (6R9-6-[N-(2-chloro-4-fluorophenyl)sulfamoyl]cyclohex-1-ene-1-carboxylate
TBK1: TANK-binding kinase 1
Tet2: Ten-eleven translocation methylcytosine dioxygenase 2
Tet2-CH: Tet2-deficient clonal hematopoiesis
TEY: Threonine-glutamate-tyrosine
TFEB: Transcription factor EB
TfR: Transferrin receptor
Th17: T helper 17
THBS1: Thrombospondin-1
TKL: Tyrosine kinase-like
tKO: Triple knockout
TLR4: Toll-like receptor 4
TLT1: TREM-like transcript 1
TNF: Tumor necrosis factor
TNFR1: TNF receptor 1
TNK1: Tyrosine kinase nonreceptor 1
Traf2: TNF receptor-associated factor 2
Treg: Regulatory T cell
TREM2: Triggering receptor expressed on myeloid cells 2
Trib1: Tribbles 1
TRIF: TIR-domain-containing adapter-inducing interferon-β
TRPM7: Transient receptor potential melastatin 7
TUNEL: Terminal deoxynucleotidyl transferase-mediated dUTP nick-end labeling
TYK2: Tyrosine kinase 2
ULK1: UNC-51-like kinase 1
VCAM-1: Vascular cell adhesion molecule 1
VLDL: Very low-density lipoprotein
WD: Western diet
WHO: World Health Organization
WT: Wildtype
ZKSCAN3: Zinc finger with krüppel-associated box and SRE-zinc finger domain associated domain domains 3
µg: Microgram
